# A proteogenomic approach for protein-level evidence of genomic variants in cancer cells

**DOI:** 10.1038/srep35305

**Published:** 2016-10-13

**Authors:** Jeonghun Yeom, Mohammad Humayun Kabir, Byungho Lim, Hee-Sung Ahn, Seon-Young Kim, Cheolju Lee

**Affiliations:** 1 Center for Theragnosis, Korea Institute of Science and Technology, Seoul 02792, Republic of Korea; 2 Department of Biological Chemistry, Korea University of Science and Technology, Daejeon 34113 Republic of Korea; 3 Genome Structure Research Center, Korea Research Institute of Bioscience and Biotechnology, Daejeon 34141, Republic of Korea; 4 Department of Functional Genomics, Korea University of Science and Technology, Daejeon 34113, Republic of Korea

## Abstract

Variations in protein coding sequence may sometimes play important roles in cancer development. However, since variants may not express into proteins due to various cellular quality control systems, it is important to get protein-level evidence of the genomic variations. We present a proteogenomic strategy getting protein-level evidence of genomic variants, which we call sequential targeted LC-MS/MS based on prediction of peptide pI and Retention time (STaLPIR). Our approach shows improved peptide identification, and has the potential for the unbiased analysis of variant sequence as well as corresponding reference sequence. Integrated analysis of DNA, mRNA and protein suggests that protein expression level of the nonsynonymous variant is regulated either before or after translation, according to influence of the variant on protein function. In conclusion, our data provides an excellent approach getting direct evidence for the expression of variant protein forms from genome sequence data.

Proteogenomics provides protein-level evidence of predicted genes and helps to improve genome annotations using integrated information of Exon/RNA sequencing and proteomic data^1^,^2^,^3^,^4^. Benefits of proteogenomics for protein information can be obtained not only for protein-coding genes, but also for novel gene models such as noncoding, mutation and fusion gene in various organisms^5^,^6^,^7^. Single nucleotide mutation in the coding region of genes cause amino acid codon alterations (nonsynonymous variants) and such alterations may lead to protein misfolding, polarity shift, improper phosphorylation and other functional consequences^8^. Recent studies have suggested signatures of mutations in various human cancers at the gene level^9^,^10^. However, identification of the mutated protein remains a highly challenging task.

The purpose of the present research is to suggest a new strategy of proteogenomics for getting protein-level evidence of genomic variations. Generally, proteomic data in proteogenomics are acquired based on shotgun proteomics, using liquid chromatography tandem mass spectrometry (LC-MS/MS)^2^,^3^,^11^. Shotgun proteomics are usually performed by data dependent acquisition method (DDA) to identify peptides. This method has a limitation in identifying target peptides from highly complex samples, due to poor peptide reproducibility and automated ion selection^12^,^13^,^14^,^15^. As an alternative to this disadvantage, various methods have been reported, such as DDA with inclusion list (Inclusion), data independent acquisition method without precursor ion selection (PAcIFIC)^16^ and differential mass spectrometry (dMS)^17^,^18^,^19^,^20^. These methods are reported beneficial in obtaining peptide spectra regardless of the intensity of precursor ion (PAcIFIC) or by giving priority to a mass of particular peptide (Inclusion and dMS). It is however difficult to apply these methods directly to test whether the genomic variations (proven by DNA sequencing) are really expressed into proteins or not. As per our observation, a critical factor behind this is the inefficiency in targeting the specific as well as relevant variant peptide sequences out of large data set. We hereby report new proteogenomic approach to address this issue by incorporating merits of previously reported methods, viz. PAcIFIC, inclusion and dMS. We named the strategy as Sequential Targeted LC-MS/MS based on Prediction of peptide pI and Retention time (STaLPIR). STaLPIR brought about increased number of identifications. Especially, the identification of the peptides that harbor the variation sites is ascertained by focusing on the genomic information-driven target peptides.

As a proof-of-concept, we present an analysis of nonsynonymous variants at the protein level by using our STaLPIR method on gastric cancer cells. Briefly, we integrated the entire exome sequence data and STaLPIR data. Subsequently, we selected a set of 296 nonsynonymous variants and confirmed the expression of 147 variants at the protein level, with further information of gene expression pattern, gene regulation and their functional aspects. Until now, despite the rise of studies on variants using proteogenomics, few have attempted to address the expressed feature of variants at the protein level. Our results provide significant information for understanding the expression of variant genes from DNA to protein, and lay a foundation for future work to treat mutant proteins that might occur in various cancers.

## Results

### Identification of nonsynonymous variation by whole-exome and RNA sequencing

**Figure 1:**
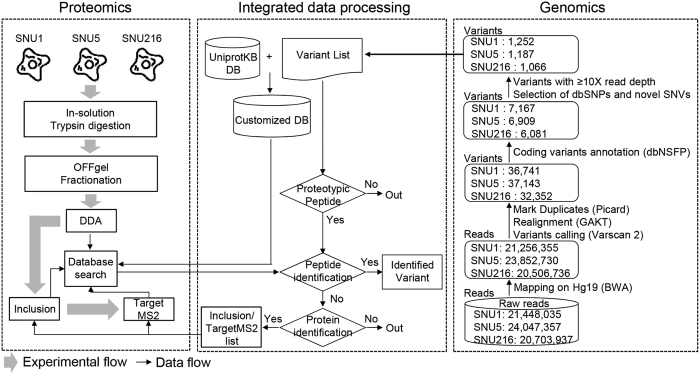
Schematic workflow of the STaLPIR proteogenomic method. The approach consists of three components: genomics, data processing and proteomics. The spectra from DDA method were searched against the customized database (UniprotKB + Variant list). Later, the RT and pI of targets were determined according to the distribution of RT’s and pI’s of identified peptides, and then Inclusion and TargetMS2 analyses were performed based on the predicted RT and pI of the targets.

To apply our proteogenomic approach to human samples, we selected three gastric cancer cell lines (SNU1, SNU5, and SNU216) as a model system, and performed both whole-exome/RNA sequencing and proteomic analysis (Fig. 1, Supplementary Methods). We expected that the lesser heterogeneous properties of cancer cell lines compared to primary tumors might facilitate straightforward interpretation of proteogenomic data. From sequencing data, we obtained a total of 2,220 variants as final sets of nonsynonymous variants, including 1,910 dbSNPs, 45 COSMIC variants, and 265 novel variants (Supplementary Fig. S1a). Of them, 379 overlapped and 1,314 unique variants were observed between the three cell lines (Supplementary Fig. S1b). The average expression level of genes harboring selected variants was 3.2 in SNU1, 3.1 in SNU5, and 3.5 in SNU216 at the Log_2_ RPKM level (Supplementary Fig. S1c, ranging from 0 to 14.6).

### Proteogenomic strategy for identification of variants at protein level

**Figure 2:**
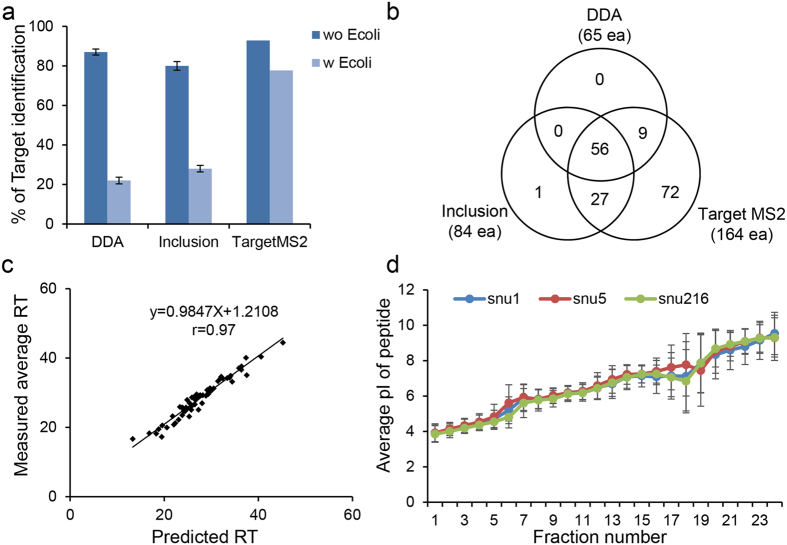
Features of STaLPIR. (**a**) 211 synthetic peptides were analyzed by three MS methods. The TargetMS2 showed the highest identification efficiency among the three MS methods when target peptides were complexed with *E. coli* digests. (**b**) TargetMS2 identified target peptides the most and covered almost all identified peptides by other methods. (**c**) The measured retention time (RT) of synthetic peptides was similar to the predicted RT. (**d**) A fair increase in pI value was observed from fraction 01 to fraction 24 of OFFGEL fractions. The upper and lower pI values for each fraction were determined from the standard deviations and, subsequently, used for Inclusion and TargetMS2 method set-up.

For efficient identification of variant proteins in a complex proteome, we have developed a genomic information-based LC-MS/MS method which we named as **s**equential **ta**rgeted **L**C-MS/MS based on **p**rediction of peptide p**I** and **R**etention time (STaLPIR) (Fig. 1, Supplementary Methods). The STaLPIR consisted of two precursor ion dependent acquisition methods (DDA, Inclusion) and one precursor ion independent acquisition method (TargetMS2). We initially evaluated the performance of the three methods using a standard peptide mixture with/without peptides from a tryptic digestion of *Escherichia coli* (*E. coli*) lysate (Supplementary Methods). The standard peptide mixture was prepared by mixing arbitrarily diluted 211 synthetic peptides and tested for DDA, Inclusion and TargetMS2 methods directly (without *E. coli*) or after spiking into *E. coli* digests (with *E. coli*). Both mixtures were run in triplicate in DDA and Inclusion methods, but only once in TargetMS2. The number of identifications for the standard mixture without *E. coli* digests was almost identical in all the three methods (Fig. 2a). The identifications were decreased for the mixture with *E. coli* digests. However, the extent of decrease was different between the methods; Target MS2 method was the most tolerant to the matrix effect and resulted in 2.6- to 3.5-fold improved peptide identification compared to DDA or Inclusion method (Fig. 2b). Notably, most of the peptides identified in the DDA and Inclusion methods were also detected in TargetMS2. These results indicate that TargetMS2 is more powerful than DDA or Inclusion in identifying pre-defined targets in a complex sample.

The number of TargetMS2 runs is determined by the number of targets. For example, we ran 11 TargetMS2 cycles to analyze 211 synthetic peptides, and analyzed an average of 19 peptides in a single run. In other words, the difficulties encountered by the TargetMS2 include requirement of larger quantity and more time to analyze samples. The STaLPIR avoids both issues by targeting the peptides neither found in DDA nor Inclusion, and by categorizing and scheduling the targets based on their retention time (RT) during RPLC and isoelectric point (pI) by which prefractionation is accomplished. First, we assessed the reproducibility of our LC-MS/MS using the standard peptide mixture with *E. coli* digests. We performed the DDA experiment at 0 hour, 16 hours and 24 hours without any intervening column-washings, and identified 29 overlapping peptides from three experiments. The relationship of RTs between each pair of experiments was highly linear (average r = 0.99) and a minor deviation (average difference = 0.1 min) was observed (Supplementary Fig. S2). A total of 65 peptides, identified in three experiments, were used to predict RTs using SSRCalc 3.0. Comparing measured RTs with predicted RTs from Fig. 2c, a high degree of linearity (r = 0.97) was observed. Second, the peptide samples from gastric cancer cell lines were fractionated according to the pI of peptides by using an OFFGEL fractionator; later, each fraction was analyzed by DDA. The average pI observed were similar in distribution in all cell lines, and the same held true for standard deviations, ranging from 0.3 to 2.0 (Fig. 2d). Based on these results, the target peptides were analyzed in fractions appropriate to pI of each peptide (Supplementary Fig. S3, Supplementary Methods). From the data mentioned above, we can conclude that STaLPIR increases the identification of pre-defined target peptides by sequential analysis based on prediction of RT and pI.

### Identification of single amino acid variations in gastric cancer cells

**Figure 3:**
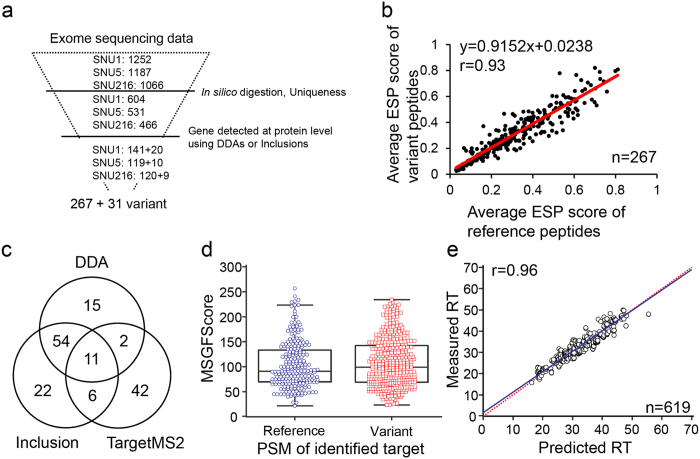
Identification of nonsynonymous variants using STaLPIR. (**a**) Number of nonsynonymous variants at each step of *in silico* analysis and STaLPIR workflow. Finally, 298 variants were detected at the protein level. The 267 variants had both reference and variant peptides, whereas 31 variants had only variant peptide. (**b**) The ESP score of selected peptides for 267 variants did not differ significantly between reference (x-axis) and variant (y-axis). (**c**) Venn diagram of the target peptides identified by different MS methods. (**d**) Distribution of database search score for all PSMs of target peptides. (**e**) The relationship between measured and predicted RTs of all PSM of target peptides.

Although the whole exome sequencing of gastric cancer cell lines identified 2,220 nonsynonymous variants, only 1,029 variants yielded unique tryptic peptides when predicted by *in silico* digestion. Again, the proteins to which 298 of 1,029 variants belonged were identified by DDA and Inclusion, even though most of the proteins were identified by those other than variant peptides. Therefore, the 298 variants were selected as targets of STaLPIR in order to detect the variant sequence-containing peptides. In addition, the corresponding reference peptides (reference sequence-containing peptides) were also targeted for STaLPIR (Supplementary Methods, Fig. 3a). The detection of each peptide does not depend only on its expression level but also the physicochemical properties in LC-MS/MS. We evaluated whether the detection probability was inadvertently perturbed by change in amino acid sequence. Here, we used the enhanced signature peptide (ESP) predictor^21^ to evaluate the probability (ESP score) of each peptide. The selected peptides have similar potential of ion-current response between reference and variant peptides (Fig. 3b). The relationship between average ESP score of reference and variant peptides was highly linear (r = 0.93), and the slope was approximately one (m = 0.9152; b = 0.0238). The analysis implies that detectability of reference and variant peptides are largely dependent on their expression level and rarely on the ionization efficiency during LC-MS/MS.

As the standard protein database does not have a variant sequence, we performed the database search of STaLPIR data with a customized database. The database consisted of UniprotKB database and variant proteins translated from whole exome sequencing of individual samples (Supplementary Methods). Finally, we confirmed protein-level expression of 147 variants (69 in SNU1, 47 in SNU5, 61 in SNU216) with 224 unique peptides, by using the MSGF+ search engine^22^ with an FDR ≤0.01 (Supplementary Table S1). Of the variant peptides, 15 were solely identified by DDA. The Inclusion method increased the number of identifications, and the maximum was achieved by TargetMS2 method, where 42 additional peptides were identified (Fig. 3c). There was not a marked contrast between the identification scores of reference and variant peptides (Fig. 3d). The measured RT of Target PSM was similar to predicted RT in three method (Fig. 3e). Altogether, the results support that our STaLPIR strategy (including DDA, Inclusion and TargetMS2) results in an improved identification of specific genomic features.

### Correlation between proteome and transcriptome data of variant genes

**Figure 4:**
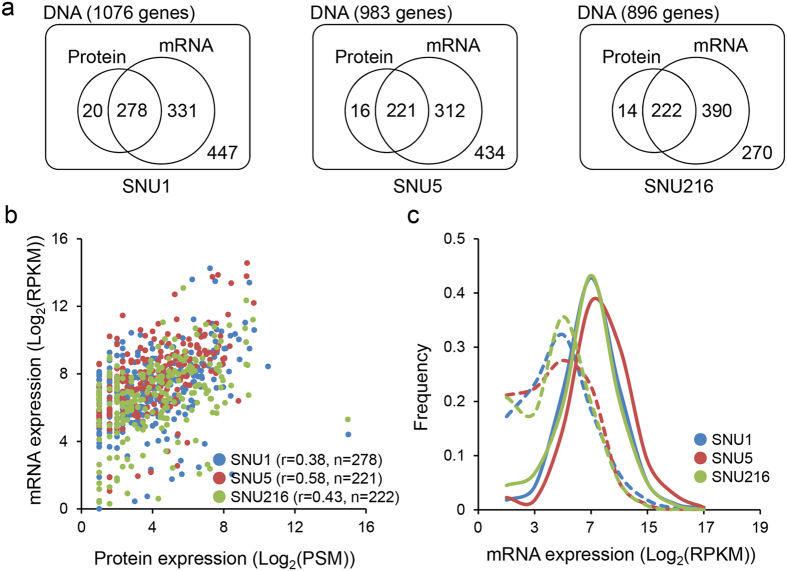
mRNA and protein levels of variant genes. (**a**) Number of variants detected at genomic, transcriptomic and proteomic levels. Average 20% variants were overlapped in both mRNA and protein levels. (**b**) Correlation of mRNA (RPKM) versus Protein (PSMs) expression levels in three gastric cancer cells. Plotted are the log2-transformed ratios. (**c**) Distribution of mRNA expression for variant gene at protein level (dashed line - not detected, solid line - detected).

The nonsynonymous variants were uncovered from 1,764 genes (1,076 in SNU1, 983 in SNU5, 896 in SNU216) using whole exome sequencing. Of these, expression of 1,153 genes were observed at the mRNA level, and 534 genes were identified at the protein level, regardless of detection of variant peptides (Fig. 4a); 20% of variant genes were detected in both mRNA and protein level. We thereafter attempted to compare protein *versus* mRNA abundance of all genes or only variant genes (Fig. 4b, Supplementary Fig. S4a). We observed a modest correlation in both cases and were unable to discover a feature specific only to the variants. In contrast, we observed differed mRNA expression level depending on the presence or absence of the protein identification. The genes detected at the protein level tended to have a higher mRNA expression level as compared to genes not detected at protein levels, in both variant genes and whole genes (Fig. 4c, Supplementary Fig. S4b). In conclusion, the correlation data is limited in making any inference about gene regulation of variant; however, most genes require the expression of more than a certain level of mRNA without difference between normal and variant genes.

### Regulation of variant gene expression

For analysis of allelic expression pattern at protein-level, not only the variant peptides but also the reference peptides were targeted for identification using STaLPIR (Supplementary Table S2). The allele expression is characterized as reference (AA), hetero (AB) and variant (BB) type. The types, as represented in this study, means ‘read count’ in case of DNA/mRNA, and the number of peptide spectra match (PSM) in case of proteins. The variant allele expression in our study can be classified according to the change in allele type of DNA to Protein as class 1, class 2 and class 3. Most of the identified belonged to class 1 (89 variants in each cell line) where expression type at the mRNA and peptide was identical (Supplementary Fig. S5a). Furthermore, we calculated the proportion of variants based on the number of sequencing reads in mRNA and PSMs at the peptide level. The correlation between peptide and mRNA was significant (0.94) in class 1. However, heterogeneous expression was observed in the class 2 (28 variants in each cell line) in both DNA and RNA, whereas their peptides were only identified in either reference or variant peptides. Also, the correlation in class 2 (0.55) was insignificant when compared to class 1. Class 3 (60 variants in each cell line) variants identified in mRNA and peptide levels were found to be quite different (Supplementary Fig. S5b). Although 70% class 3 variants differed between mRNA and peptide, the allele type of peptide level was identical with the DNA level. Assuming the absence of technical and computational noise, class 3 is presumed to be regulated through a very complex process in transcription or post-transcription. The newly discovered class 3 variants remain as a matter, and needs to be further discussed. The allele type expression in class 1 and class 2 seem to be differently regulated. In addition, the functional categories of class 1 genes were enriched in polymorphism (86.4%) using DAVID Bioinformatics Resource^23^. Again, 75% of class 2 was enriched to phosphoproteins which are post-translationally modified. Class 3 was similar to class 1 (Supplementary Fig. S5c). Cellular component analysis indicated that each class showed enrichment in nucleolus, ribonucleoprotein complex and mitochondrion, respectively (Supplementary Fig. S5d). There are some hints, which might suggest the possibility that the allele of class 1 was expressed under transcriptional regulation and the allele of class 2 was expressed under post-transcriptional regulation. We confirmed seven variants in whole cell lysate using multiple reaction monitoring mass spectrometry (Supplementary Methods). The pattern of allele type expression was similar to our STaLPIR results (Supplementary Fig. S6).

### Relationship between the features of variation and protein-level expression

There are six variations of nucleotide pair (C:G > A:T, C:G > G:C, C:G > T:A, T:A > A:T, T:A > C:G and T:A > G:C). The distribution of frequency versus variation type, did not change significantly by the cell type or dataset (Supplementary Fig. S7). Our result bears an interesting pattern of mutational signatures related to stomach cancer shown in a recent study^9^. According to the study, 12 signature types prevail in stomach cancers and most of these features are represented by high frequency of C:G > T:A type. Especially, “signature 20” is associated with defective DNA mismatch repair, which is similar to our result that showed enrichment mostly at C:G > A:T and C:G > T:A. Evaluating the findings of variation pattern, it can be inferred that the identified variants are disease-specifically related to stomach cancer.

We also analyzed the possible functional effects of variations. We used *in silico* algorithms such as MutationAssessor^24^, SIFT^25^,^26^,^27^,^28^ and PolyPhen-2^29^ to predict the functional impact of identified variations on proteins. MutationAssessor considers disease-associated with scores ≥1.938 and SIFT considers predictive of intolerant changes with score ≤0.05, whereas PolyPhen-2 considers mutation with score ≥0.453 as damaging. We observed a difference of such scores in accordance with allele type at protein level. BB type was expected as less affected than AA type, whereas AB type was predicted with intermediate score between AA and BB type (Fig. 4). Besides, the functional annotation of each allele type was performed using DAVID (Supplementary Table S3). The results with P < 0.05 were visualized and displayed as a network by Cytoscape (Supplementary Fig. S8)^30^. In AA type, the significantly enriched term was “acetylation”, which occurs as a co-translational and post-translational modification of proteins. In contrast, BB type-enriched terms were “polymorphism” and “sequence variant”, which are related to biodiversity. AB type-enriched terms were “acetylation” and “sequence variant”. Thus, we infer that the variations of AA type prevented the expression of mutated proteins due to intracellular quality control, and the mutated proteins of BB type were well expressed like reference proteins. BB-type variants that do not follow the general characters of BB-type, for example, those not classified as polymorphic and having variations of high impact score, may be good candidates of gastric cancer-specific disease proteins.

## Discussion

This study proposes a comprehensive proteogenomic approach in mapping of nonsynonymous variants at the protein level. Furthermore, our study provides not only the identification of nonsynonymous variants at the protein level, but also the information about expression pattern, relationship between mRNA and protein, pattern of allele type and functional impact of variant. To identify variant, we developed a new proteogenomic approach (STaLPIR) with improvement in peptide identification. Nevertheless, there is a limitation to this study, which needs to be further explored. We identified 2224 variant from the genomic data, but only ~13% (298) were selected as targets. The presence of a unique tryptic peptide is essential for proteomic analysis. Some variants had changes to the amino acid of trypsin cleavage site such as K or R, and some changes were isobaric like I > L or L > I. In other cases, the tryptic peptide where the variation occurred was not unique and could not be distinguished from the peptides of other proteins. Therefore, it requires further analysis employing multiple proteases with different cleavage specificities.

**Figure 5:**
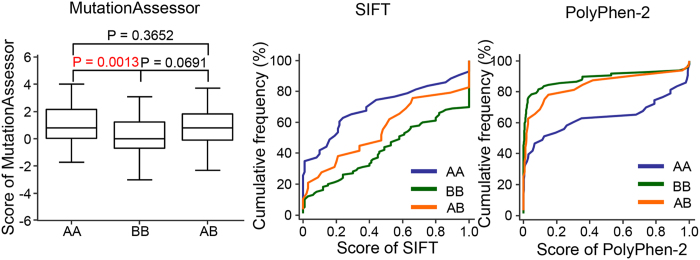
Comparison of functional impact based on allelic expression pattern at protein level. Three independent algorithms (MutationAssessor, SIFT and PolyPhen-2) were used. All identified variants were classified as AA, BB, AB type. “A” refers the reference sequences found at the protein level, “B” refers the variant sequences found at the protein level.

We considered allele types of variations at DNA, mRNA and protein levels for analysis of allele type change during gene expression. Our STaLPIR approach is suitable for such analysis because not only the variant form but also its reference form can be targeted in TargetMS2 runs, regardless of the read count of each allele type at the genomic level. We speculate that the expression level of variant proteins are regulated either before or after translation. Especially, our study revealed that the difference of protein levels between allele types was clearly depicted by the functional impact of variations that discerned the allele types (Fig. 5). In eukaryotes, the quality control system of protein synthesis, such as nonsense-mediated decay^31^,^32^ and ubiquitin-proteasome system^33^, prevents the expression of aberrant genes. Our findings suggest that if a nonsynonymous variant affects the protein function severely, protein synthesis of this variant is regulated by the cellular quality control system or surveillance pathway, and therefore wild type allele prevails in expression.

Although our present study has focused on identification of nonsynonymous variant proteins, STaLPIR is also applicable to study for the identification of other genomic aberrations such as fusion gene, novel splice junction, insertions and deletions. These application studies also need to consider the presence of unique peptides corresponding to the aberration sites at the genomic loci. Subsequently, the novel peptides of interest are targeted by STaLPIR to get protein-level evidence of such genomic aberrations. In conclusion, our result provides an outstanding approach by which we can obtain evidence for the expression of variants at the protein level, and helps to understand the relationship between gene and protein.

## Methods

### Cells

Three gastric cancer cell lines, SNU1, SNU5 and SNU216 were used throughout the study. The cells were grown in Gibco^®^ RPMI 1640 medium (Life Technologies, Carlsbad, CA) supplemented with 10% fetal-bovine serum (FBS; Gibco^®^), 100 units/mL penicillin and 100 μg/mL streptomycin.

### Whole exome and RNA sequencing

The genomic DNAs were isolated from the cells using the PuregeneTM DNA purification kit (Qiagen). Library construction and exome enrichment were performed using Illumina TruSeq DNA Sample Prep Kit-Set A, SeqCap EZ Human Exome Library v2.0, and SeqCap EZ hyb and wash kit (Roche NimbleGen). Next, exome enriched libraries were sequenced using Illumina Genome Analyzer IIx. The resulting sequencing reads were aligned on human reference genome 19 (hg19) with Burrows Wheelers Aligner (BWA)^34^. VarScan 2 was used to identify genetic variants by comparing genome sequences with hg19^35^ and dbNSFP was used to annotate protein coding variants^36^. Identified genetic variants with more than 10X coverage were selected for subsequent analyses. Total RNAs were extracted using RNeasy^®^ Mini kit (Qiagen). Libraries were constructed using Illumina TruSeq RNA Sample Prep Kit v2-Set A and sequencing was carried out using the Illumina Genome Analyzer IIx. Following this, the reads were mapped on hg19 using TopHat v2.0.6^37^, and the expression level of transcripts was estimated using our customized Python code, which calculates the reads per kilobase per million reads (RPKM).

### Extraction, digestion, fractionation and mass spectrometric analysis of proteins

Proteins were extracted from the three gastric cancer cell lines and proteolyzed using trypsin. The resultant peptides were separated into 24 fractions according to their pI on an Agilent 3100 OFFGEL Fractionator equipped with pH 3–10 linear IPG strips (24 cm, GE Healthcare). Next, peptides in each fraction were loaded onto a reversed-phase Magic C18aq (Michrom BioResources, Auburn, CA) column (15 cm × 75 μm, packed in-house) on an Eksigent nanoLC-ultra 1D plus system, and eluted with a linear gradient from 5% to 30% acetonitrile in 0.1% formic acid at a flow rate of 300 nL/min. The eluted peptides were analyzed using Q Exactive quadrupole mass spectrometer (Thermo Scientific, Bremen, Germany) operating in a data-dependent acquisition mode with (referred to as Inclusion) or without (referred to as DDA) an inclusion list or TargetMS2 mode. In DDA and Inclusion modes, survey full-scan MS spectra (m/z 400–1,800) were acquired with a resolution of 70,000. The MS/MS spectra of the 12 most intense ions from the MS1 scan with a charge state ≥2 were acquired with the following parameters: resolution, 17,500; automatic gain control (AGC) target, 1E5; isolation width, 2.0 m/z; normalized collision energy, 27%; dynamic exclusion duration, 30 s; and ion selection threshold, 4.00E + 03 counts. In TargetMS2 mode, MS/MS spectra were directly acquired without survey full scans. The TargetMS2 mode was operated at similar parameters with the exception of 2E5 for AGC target.

### Database search and processing of proteomic data

We first constructed customized protein databases by appending human UniprotKB database (released May 2013) with the amino acid sequences of nonsynonymous variants identified by whole exome sequencing. MS2 spectra obtained from DDA, Inclusion and TargetMS2 experiments were searched against our customized databases using MSGF+ search engine with following settings: number of tolerable termini = 1; MS1 tolerance = 15 ppm; isotopeErrorRange = 0,2; and allowance of decoy database search. The false discovery rate (FDR) was set to 1% at PSM level. For protein assembly, search output files from DDA and Inclusion were submitted to IDPicker 3.1. Here, for further refinement of data, FDR at spectrum, peptide and protein level were set to ≤1% with minimum of 2 unique peptides and spectra.

### Generation of target peptide list for Inclusion and TargetMS2

The sequences of reference and variant proteins were *in silico* digested using trypsin with the following options: two missed cleavages; full tryptic digestion and mass (MH+) range (600–4,000). Subsequently, the tryptic peptides containing variation sites were picked up and filtered against the following parameters: peptide length (8–25 amino acids); pI value (3–10.99) and no N-terminal cysteine (C), glutamine (Q) and glutamic acid (E). We arranged the target peptides as per pI value, and classified them into 24 OFFGEL fraction groups. Finally, the Inclusion and TargetMS2 lists were prepared by Skyline software (version 2.5)^38^ with retention time that was predicted by SSRCalC 3.0. Maximum concurrent precursors for TargetMS2 was set to 20 with 10-minute time window.

### Measurement of variant peptides by LC-MRM

Variant peptides as well as the corresponding reference peptides were measured by LC-MRM-MS on a 5500 Qtrap mass spectrometer equipped with an Eksigent nanoLC-Ultra 2D plus and a nanoelectrospray ion source (SCIEX, Foster City, CA, USA). The instrument was operated in positive mode with the following parameters: ion spray voltage of 2,100 V, curtain gas at 20 psi, nebulizer gas at 25 psi, resolution at 0.7 Da (unit resolution) for Q1 and Q3, interface temperature at 150 °C, and scan mass range of 300–1,250 m/z. The collisional energy (CE), collisional cell exit potential (CXP) and declustering potential (DP) were optimized for each peptide by direct infusion using Turbospray. Quantification experiments were performed using a scheduled LC-MRM mode with MRM detection window of 480 s and cycle time of 1.5 s. Before analysis, samples were spiked with 400 fmol of digested *E. coli* β-galactosidase (#4465938, SCIEX). Skyline was used to generate extracted ion chromatograms (XICs) of target peptides. Then, peak areas (MRM signals) of XICs were normalized against the geometric mean of the peak areas of two β-galactosidase peptides (FNDDFSR and LNVENPK).

### Bioinformatic analysis

ESP Predictor (http://www.broadinstitute.org/cancer/software/genepattern/esppredictor) was used to predict ionization efficiency of STaLPIR peptides during mass spectrometry. For functional annotation of proteins, DAVID (Database for Annotation, Visualization and Integrated Discovery, http://david.abcc.ncifcrf.gov/) was used (Huang da *et al*. 2009). Functional annotation charts were generated using each of the following GO terms: Biological Process, Molecular Function, Cellular Component, Uniprot Sequence Feature, and Protein Information Resource. Results were considered significant at P-value < 0.05 and visualized by the Enrichment Map plugin Cytoscape.

## Additional Information

**How to cite this article**: Yeom, J. *et al*. A proteogenomic approach for protein-level evidence of genomic variants in cancer cells. *Sci. Rep.* **6**, 35305; doi: 10.1038/srep35305 (2016).

## Supplementary Material

Supplementary Information
