# Supplementary material for: A proteogenomic approach for protein-level evidence of genomic variants in cancer cells
Source: Sci Rep. 2016 Oct 13;6:35305. doi: 10.1038/srep35305 (PMC5062161; doi:10.1038/srep35305)
Supplement: Supplementary Information [file srep35305-s1.pdf]

Supplementary Information

A proteogenomic approach for protein-level evidence of genomic variants in cancer cells

Jeonghun Yeom[1,2], Mohammad Humayun Kabir[1], Byungho Lim[3], Hee-Sung Ahn[1,2], Seon-Young Kim[3,4], Cheolju Lee[1,2]

[1]Center for Theragnosis, Korea Institute of Science and Technology, Seoul 02792, Republic of Korea

[2]Department of Biological Chemistry, Korea University of Science and Technology, Daejeon 34113 Republic of Korea

[3]Genome Structure Research Center, Korea Research Institute of Bioscience and Biotechnology, Daejeon 34141, Republic of Korea

[4]Department of Functional Genomics, Korea University of Science and Technology, Daejeon 34113, Republic of Korea

Corresponding author: Cheolju Lee. Phone: +82-2-958-6788; E-mail: clee270@kist.re.kr.

Supplementary Methods

Materials

Protease inhibitor cocktail was obtained from Roche (Mannheim, Germany). Sequencing-grade modified trypsin was purchased from Promega (Madison, WI). Tris(2-carboxyethyl)phosphine hydrochloride (TCEP-HCl) was purchased from Thermo (Thermo Scientific, Rockford, IL) and Dithiothreitol (DTT) was from Sigma (Sigma–Aldrich, Inc., St. Louis, MO). All other chemicals and reagents used were of the highest grade commercially available. Crude synthetic peptides (211 peptides) used for LC-MS/MS optimization were from the remnants of our previous study [39]. Peptides stored in a fridge at ~250 nmol/mL in 20% acetonitrile and 1% formic acid were arbitrarily diluted and mixed. Crude synthetic peptides (24 peptides) used for LC-MRM-MS were purchased from JPT Peptide Technologies (Berlin, Germany)

Cell culture and lysis

SNU1, SNU5 and SNU216 cell lines were grown in Gibco® RPMI 1640 medium (Life Technologies, Carlsbad, CA) supplemented with 10% fetal-bovine serum (FBS; Gibco®), 100 units/mL penicillin and 100 µg/mL streptomycin. Cultures were maintained at an atmosphere of 5% CO_2 and 95% air in a humidified incubator at 37°C. Adherent cultures were passaged at sub-confluence after trypsinization. Cells were grown around 70–80% confluence. After growing and before harvesting, cells were washed twice with ice cold phosphate buffered saline (PBS). *E. coli* DH5α was grown 37°C overnight on Lurea-Bertani (LB, 1% tryptone, 0.5% yeast extreact, 1% NaCl) medium. The gastric cancer cells and *E. coli* cell were harvested by centrifugation and then resuspended in ice cold lysis buffer containing 8 M urea, 75 mM NaCl, 50 mM Tris–Cl (pH 7.5) and a protease inhibitor mix. After 10 min incubation on ice, cells were lysed by sonication. Cell debris were removed by centrifugation at 2,500 ×*g* for 10 min at 4°C. The supernatant was centrifuged one more time for 10 min at the same speed to remove any remaining small particles. Finally, supernatant

was collected and stored at −80 °C until use. Protein concentration was determined by using a micro BCA kit (Thermo Fisher Scientific Inc., Rockford, IL).

Whole exome sequencing

The genomic DNAs were isolated from SNU1, SNU5, and SNU216 gastric cancer cell lines using the PuregeneTM DNA purification kit (Qiagen). Library construction and exome enrichment were performed using Illumina TruSeq DNA Sample Prep Kit-Set A, SeqCap EZ Human Exome Library v2.0, and SeqCap EZ hyb and wash kit (Roche NimbleGen) according to the manufacturer's instructions. Briefly, purified genomic DNAs were sheared using the Covaris S2 sonicator (Covaris). The fragmented DNAs were end-repaired and ligated with adapters. Next, exome enriched libraries were sequenced using Illumina Genome Analyzer IIx according to the manufacturer's instructions. The resulting sequencing reads were processed by bioinformatic analyses as presented in Fig. 1. Briefly, sequencing reads were aligned on human reference genome 19 (hg19) with Burrows Wheelers Aligner (BWA) and duplicates were removed by Picard. GATK realigner was used to obtain realigned BAM files [40]. VarScan 2 was used to identify genetic variants of SNU1, SNU5, and SNU216 cell lines by comparing genome sequences with hg19. To obtain protein coding variants for proteomics, dbNSFP was used to annotate coding variants. For further subsequent analyses, Identified genetic variants with more than 10X coverage were selected.

Digestion of proteins

Proteins extracted from each cell line (1 mg/mL in lysis buffer) were reduced in 5 mM TCEP for 60 minutes at 25°C. Later, cysteines were alkylated using iodoacetamide at a final concentration of 15 mM for 60 min at 25°C in the dark. Subsequently, samples were diluted 10-fold with 50 mM Tris-HCl to reduce the concentration of urea to 0.8 M. The protein sample was digested using trypsin at a

1:50 enzyme-substrate ratio for overnight at 37°C. The digested sample was allowed to cool at room temperature and digestion was quenched by acidification with trifluoroacetic acid (TFA) at a 0.5% final concentration. The sample was subsequently purified/desalted using C18 MacroSpin column (The Nest Group Inc., Southborough, MA), divided into two parts (10% for LC calibration and 90% for further fractionation), dried *in vacuo* and stored at −20 °C until further use. Digestion of *E. coli* proteins was carried out at the same manner as gastric cancer cells.

Fractionation of peptides based on isoelectric point

Peptides were fractionated according to their pI on an Agilent 3100 OFFGEL Fractionator with an optimized protocol using consumables from Agilent. Briefly, IPG strips (pH 3-10, linear, 24 cm, GE Healthcare) were inserted into off-gel trays, fixed with frames, which had been clipped to 24 wells, and rehydrated using 40 µL of peptide IPG strip rehydration solution per well for 15 min at 25°C. Peptides to run in each IPG strips were diluted in 3,600 µL of OFFGEL peptide sample solution and applied to the strip at 150 µL per well. Isoelectric focusing was conducted at 25°C with the settings of 4,500 V, 50 µA and 200 mW until 20 kVh was reached. Fractionated samples from each well were collected in individual microtube and acidified with 0.1% TFA before desalting with Pierce® C18 spin column (Thermo Scientific, Rockford, IL).

Liquid Chromatography Mass Spectrometry (LC-MS/MS)

Dried peptides were diluted with 0.4% acetic acid to attain a concentration of 1 µg/µL and an aliquot containing approximately 1µg was injected from a cooled (10 °C) autosampler into a reversed-phase Magic C18aq (Michrom BioResources, Auburn, CA) column (15 cm × 75 µm, packed in-house) on an Eksigent nanoLC-ultra 1D plus system at a flow rate of 300 nL/min. Prior to use, the column was equilibrated with 95% buffer A (0.1% formic acid in water) and 5% buffer B (0.1%

formic acid in acetonitrile). The peptides were eluted with a linear gradient from 5% to 30% buffer B over 30 min and 30% to 50% buffer B over 8 min followed by an organic wash and aqueous re-equilibration at a flow rate of 300 nL/min with a total run time of 75 minutes. The HPLC system was coupled to a Q Exactive quadrupole mass spectrometer (Thermo Scientific, Bremen, Germany) operated in the data-dependent acquisition mode or TargetMS2 mode. Data-dependent acquisition mode was operated with (referred to as Inclusion) or without (referred to as DDA) an inclusion list. Survey full-scan MS spectra (m/z 400 - 1,800) were acquired with a resolution of 70,000. Source ionization parameters were as follows: spray voltage, 1.9 kV; capillary temperature, 275 °C; and s-lens level, 44.0. The MS/MS spectra of the 12 most intense ions from the MS1 scan with a charge state ≥ 2 were acquired with the following options: resolution, 17,500; automatic gain control (AGC) target, 1E5; isolation width, 2.0 m/z; normalized collision energy, 27%; dynamic exclusion duration, 30 s; and ion selection threshold, 4.00E+03 counts. TargetMS2 was operated with similar parameters with the exception of 2E5 for AGC target. All MS runs of TargetMS2 were performed at the same condition throughout all gastric cancer cells and samples with pre-optimized of LC condition.

Database Search and data process

For matching MS/MS spectra to peptide sequences, a database search was performed using the Proteome Discoverer 1.4 software containing the Sequest algorithm (Thermo Scientific Inc., San Jose, CA). The method outline used in the spectrum selector node of the Proteome Discoverer software included all default settings. The Min and Max precursor mass settings were 350 and 5000 Da, respectively. The search parameters included were: full tryptic peptide cleavage specificity, two missed cleavages, a fixed modification of carbamidomethyl cysteine (+57.021 Da), and a variable modification of oxidized methionine (+15.995 Da). The Sequest algorithm parameters were: Dta generation threshold =10,000, precursor ion mass tolerance = 15 ppm, and fragment ion mass tolerance for ion trap ms/ms = 0.05 Da. The criteria used for acceptance of peptide assignments were as follows: Minimal XCorr value for each charged state ranging from 1 to 7 was 1.50, 2.0, 2.25, 2.75,

3.0, 3.2, and 3.4 respectively. A strict FDR of 0.01 and a relaxed FDR of 0.05, a total of two target values for a decoy database search were applied. Human UniprotKB database (May 2013) was used in all database searches except for the sample of synthetic peptides with *E. coli* digests where the human database concatenated with *E. coli* UniprotKB database (October 2013) was used.

For identification of variant peptides in gastric cancer cells. The MS raw files were converted into mzXML-files by use of ReAdW 4.3.1 (http://tools.proteomecenter.org/wiki/index.php?title=Software:ReAdW) with centroid option. Peptide and protein identification was accomplished by comparing the mzXML files with customized databases on an MSGF+ (v9881) search engine. In order to construct databases individually customized to each of three cell lines, we in-silico translated variation-containing coding sequences of whole exome sequencing data and concatenated them to human UniprotKB database (released May 2013). The search engine setting were as follows: semitrypsin; 15 ppm for MS1 mass tolerance; option #3 for instrument method; 0,2 for isotopeErrorRange; allowance of decoy database search; variable modifications: oxidation of methionine (+15.9949 Da), fixed modification: carbamidomethyl of cysteine (+57.0215 Da). The false discovery rate (FDR) was set to 1% at the PSMs level. For protein assembly, Search output files from DDA and Inclusion method were converted to IDPicker 3.1 index files using IDPicker 3.1. The FDRs of spectrum, peptide and protein were set to $\leq$ 1% with a minimum of 2 unique peptides and spectra.

Target peptide list for STaLPIR

For each of the gastric cancer cell lines, variant protein sequences were obtained from the whole exome sequence data. The sequences were *in silico* digested with trypsin. Subsequently, variant peptides, that is, the tryptic peptides containing variation sites were picked up. The same treatment was performed to reference protein sequences. The gathered peptides were filtered against the following parameters: peptide length (8 - 25 amino acids); pI value (3 - 10.99); two missed cleavages; full tryptic digestion; mass (MH+) range (600 - 4,000). Peptides containing N-terminal cysteine (C),

glutamine (Q) and glutamic acid (E) were excluded from the target list as trypsin digestion might cause artificial modification like carbamidomethylation, carboxylation, glutamine cyclization or pyroglutamate formation. The target list was used for Inclusion and TargetMS2. Initially, we arranged the target peptides as per pI value, and classified them into 24 OFFGEL fraction groups; each group has its own average pI value ± standard deviation as determined with the pI values of peptides identified from DDA. Each target peptide could be affiliated to more than one OFFGEL fraction groups whenever its pI value matched to the pI range of OFFGEL fractions. Finally, the Inclusion and TargetMS2 lists were completed by Skyline software (version 2.5) with retention time that was predicted by SSRCalC 3.0 in Skyline. Maximum concurrent precursors for TargetMS2 was set to 20 with 10-minute time window.

LC-MRM-MS setup and optimization

The LC-MRM-MS measurements were performed with two switchable columns on a nanoLC-MRM-MS system. For chromatographic separation, an Eksigent nanoLC-Ultra 2D plus interfaced with NanoFlex system (Eksigent Technologies, Redwood, CA, USA) was used. Samples were reconstituted with 20 µL of 2% acetonitrile and 0.1% formic acid, injected with a full sample loop injection of 1 µL, and separated in Nano cHiPLC ReproSil-Pur C18- columns (75 µm i.d, 15 cm length, pore size 120 Å, particle size 3 µm; Eksigent Technologies). The column was priory equilibrated with 95% mobile phase A (0.1% formic acid in water) and 5% mobile phase B (0.1% formic acid in acetonitrile). Peptides were eluted with a gradient of 5-10% mobile phase B for 4 min, 10-25% for 30 min, 25-60% for 3 min, 60%-60% for 3 min, 60-5% for 1 min, 5%-5% for 9 min at a flow rate of 300 nL/min. The LC system was coupled to a 5500 Qtrap mass spectrometer by a nanoelectrospray ion source (SCIEX, Foster City, CA, USA). The MS was operated in positive mode with the following parameters: ion spray voltage of 2,100 V, curtain gas at 20 psi, nebulizer gas at 25 psi, resolution at 0.7 Da (unit resolution) for Q1 and Q3, interface temperature at 150 °C, and scan mass range of 300 - 1,250 m/z. The collisional energy (CE), collisional cell exit potential (CXP) and

declustering potential (DP) were optimized by direct infusion using Turbospray. Quantification experiments were performed using a scheduled LC-MRM mode with MRM detection window of 480 s and cycle time of 1.5 s.

Qualification and quantification of variant peptides by LC-MRM

Skyline was applied to analyze extracted ion chromatograms (XICs) which had been imported from raw files (*.wiff) by using Analyst software (version 1.5.1, SCIEX). Crude synthetic peptides purchased from JPT Peptide Technologies were used to estimate ionization efficiency of 24 MRM targets. Two mixture stocks were prepared in 2% acetonitrile and 0.1% formic acid: the first one was the mixture of all 24 peptides at the concentration of 6.38~6.96 pmol/μL and the second one was 2.5-fold diluent of the first one. To 25 μL of the stocks, 400 fmol of digested E. coli β-galactosidase (#4465938, SCIEX) was added prior to use. LC-MRM-MS was performed three times for each of the stocks. We normalized MRM signals by dividing the raw peak area of the most intense single transition for target peptides by the geometric mean of the raw peak areas of two β-galactosidase peptides (FNDDFSR and LNVENPK). The normalized MRM signals (normalized peak area per mole) were used as scaling factors to get rough estimation of endogenous peptides in gastric cancer cells (Supplementary Table S4).

To monitor endogenous peptides in SNU1, SNU5, and SNU216 cell lysates, we performed triplicated 8-min-scheduled LC-MRM-MS. During quantification, we removed non-reliable peaks for which average peak areas were below 10,000 or CVs of triplicated runs were below 25%. Then the amount of target peptide was estimated as

$$\widetilde{M_{i,s}} = \frac{\widetilde{I_{i,s}}}{SF_i}$$

where $\widetilde{M_{i,s}}$ is the estimated mole of the i-th transition in sample s, $\widetilde{I_{i,s}}$ is i-th normalized peak area in sample s and SF_i is the scaling factor of i-th transition. All statistical data was analyzed byusing Excel 2010 (version 14.0, Microsoft Office).

Supplementary References

39. Kim, J.S. et al. Multiple reaction monitoring of multiple low-abundance transcription factors in whole lung cancer cell lysates. *J. Proteome Res.* **12**, 2582-2596 (2013).

40. McKenna, A. et al. The Genome Analysis Toolkit: a MapReduce framework for analyzing next-generation DNA sequencing data. *Genome Res.* **20**, 1297-1303 (2010).

Supplementary Figures

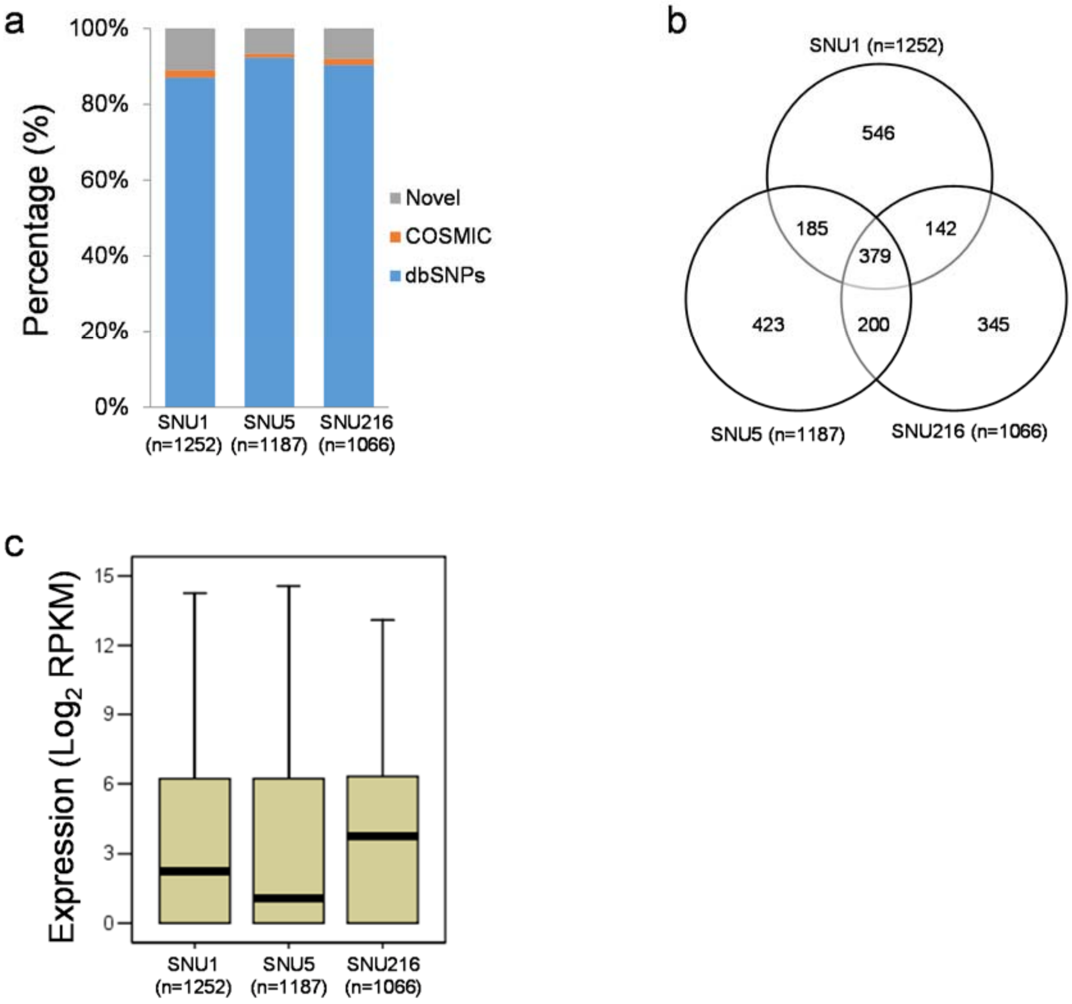

Supplementary Figure S1 Distribution of whole exome sequencing data. (a) Most of the variants match to dbSNP or COSMIC database; ~10% have not been found previously in public DB. (b) Venn diagram of the variants identified from the three gastric cancer cell lines. (c) Distribution of mRNA expression (RPKM) for variants is similar among three cell lines.

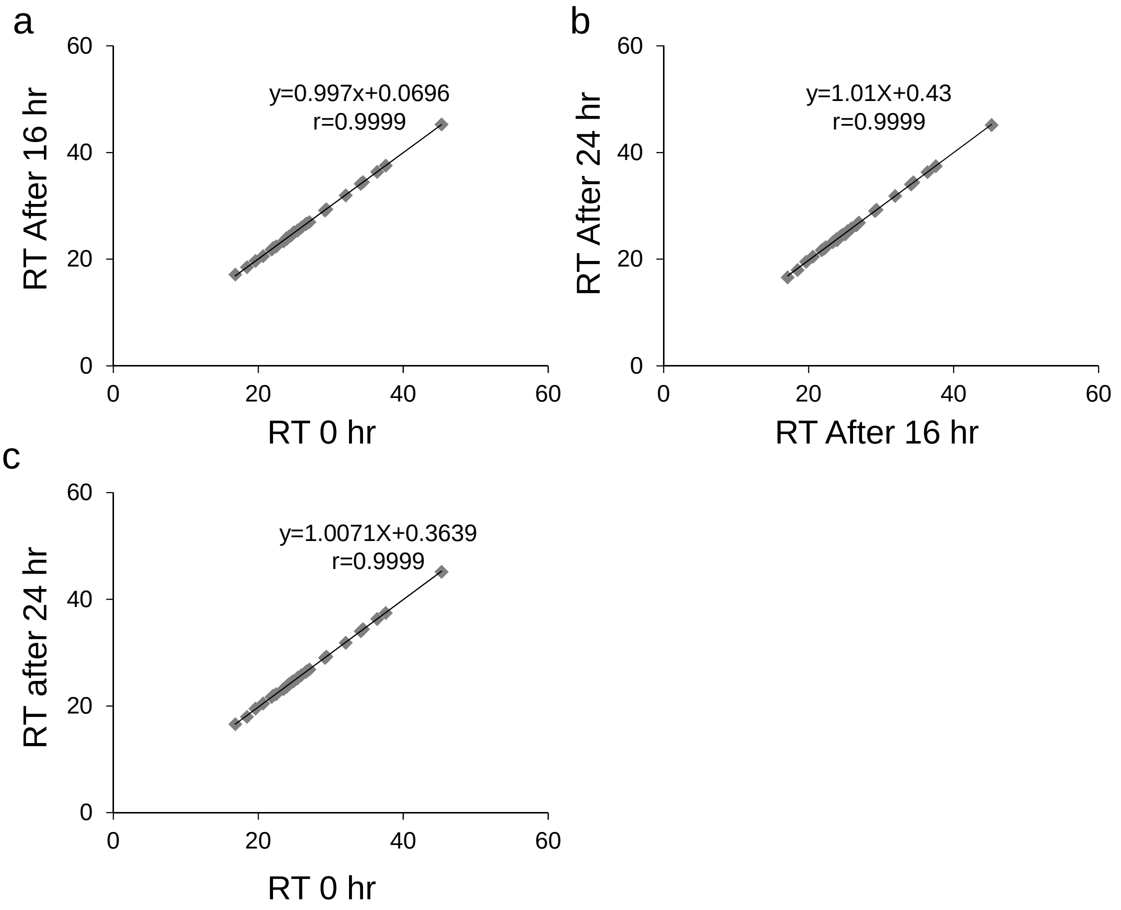

Supplementary Figure S2 Comparison of retention time of synthetic peptides at different analysis times. The relationship of RTs between each experiment is highly linear.

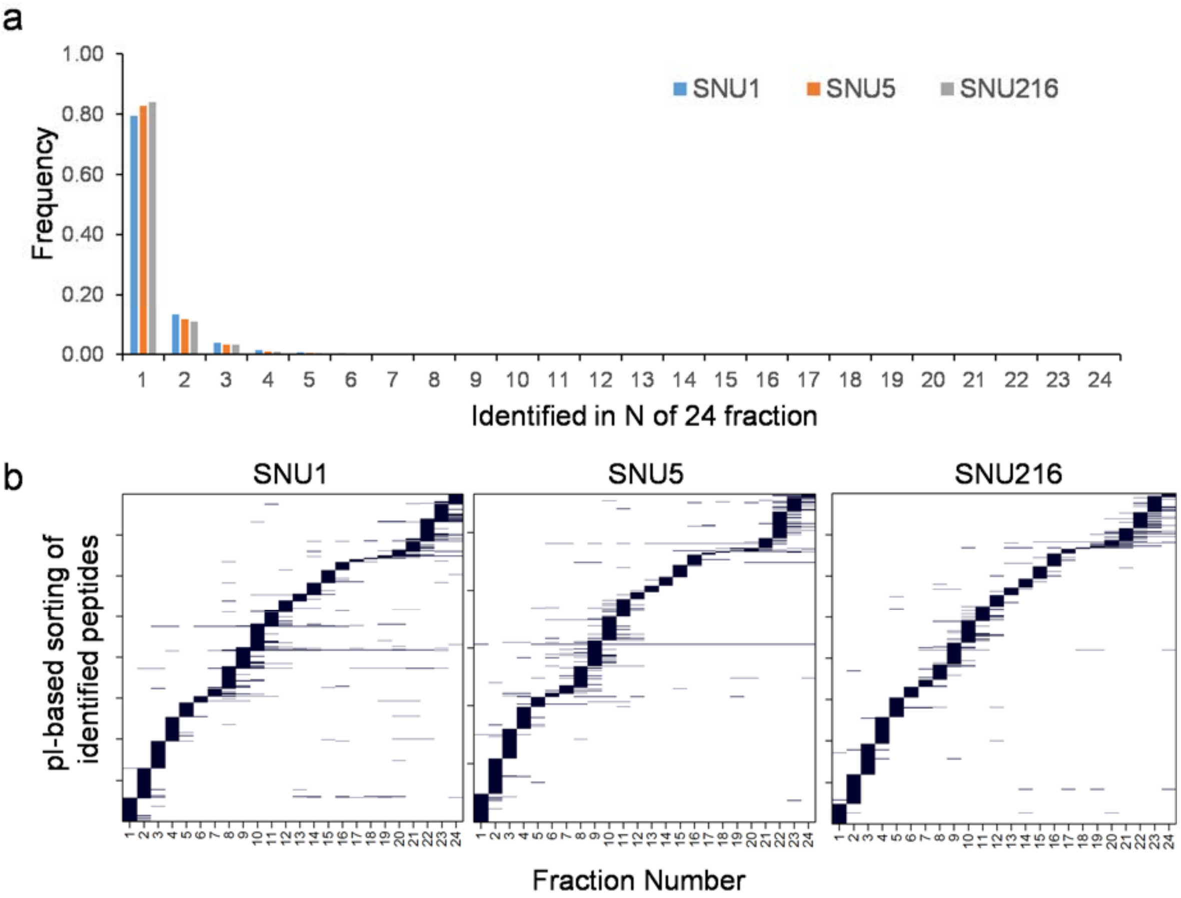

Supplementary Figure S3 Fractionwise distribution of all identified peptides from OFFGEL fractionation. (a) More than 97% peptides in each cell line are identified in 1 to 3 fractions. (b) The distribution of all peptides identified in each fraction, with the X-axis representing the fraction number and the Y-axis, the matched peptides sorted by pI in fraction.

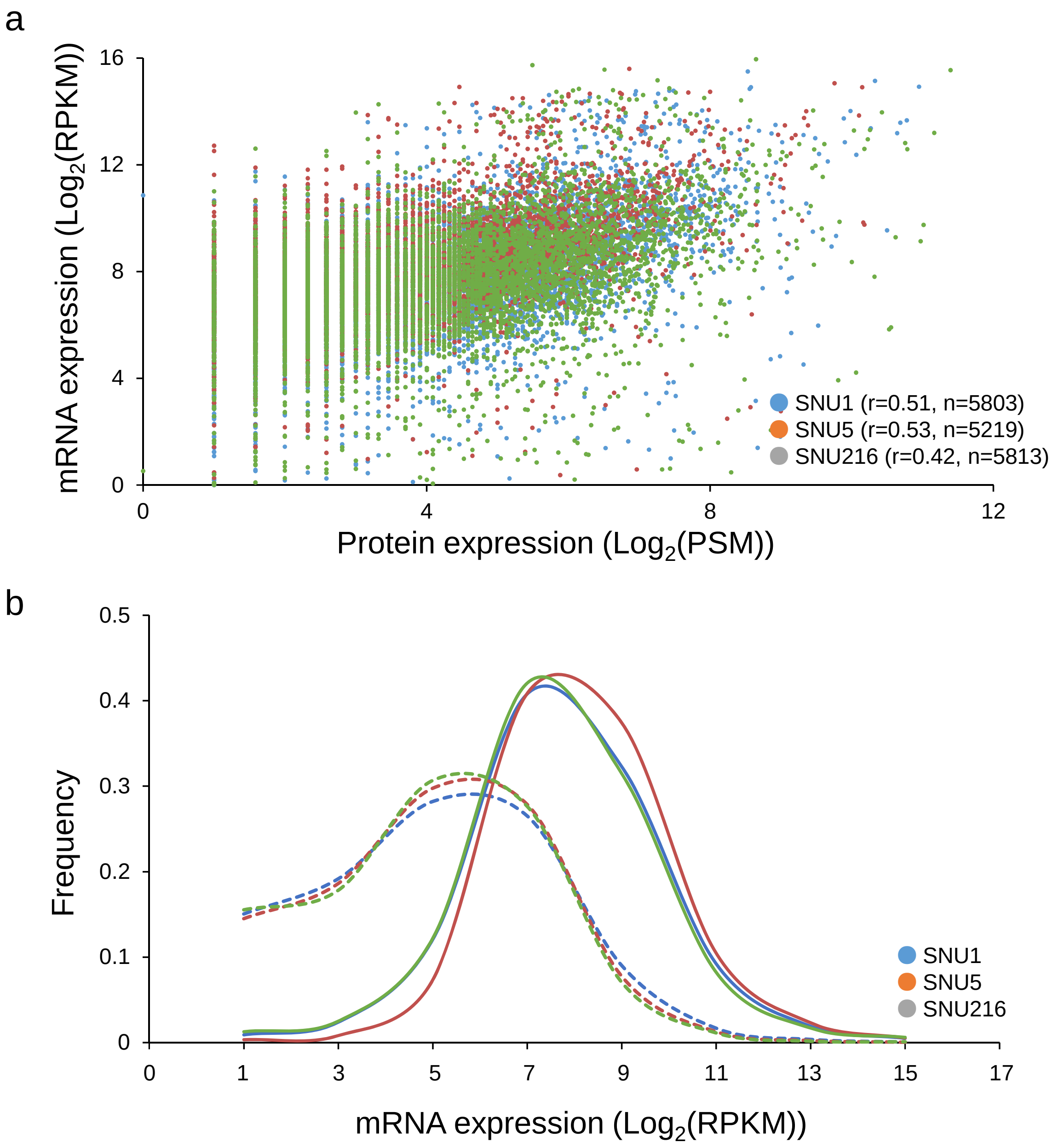

Supplementary Figure S4 Global analysis of all genes from the three gastric cancer cell lines. (a) correlation of mRNA (RPKM) versus protein (PSMs) expression levels. Pearson's correlation coefficient of all genes was similar to that of variant genes only. (b) Distribution of mRNA expression levels for all genes (dashed line, not detected at protein level; solid line, detected at protein level).

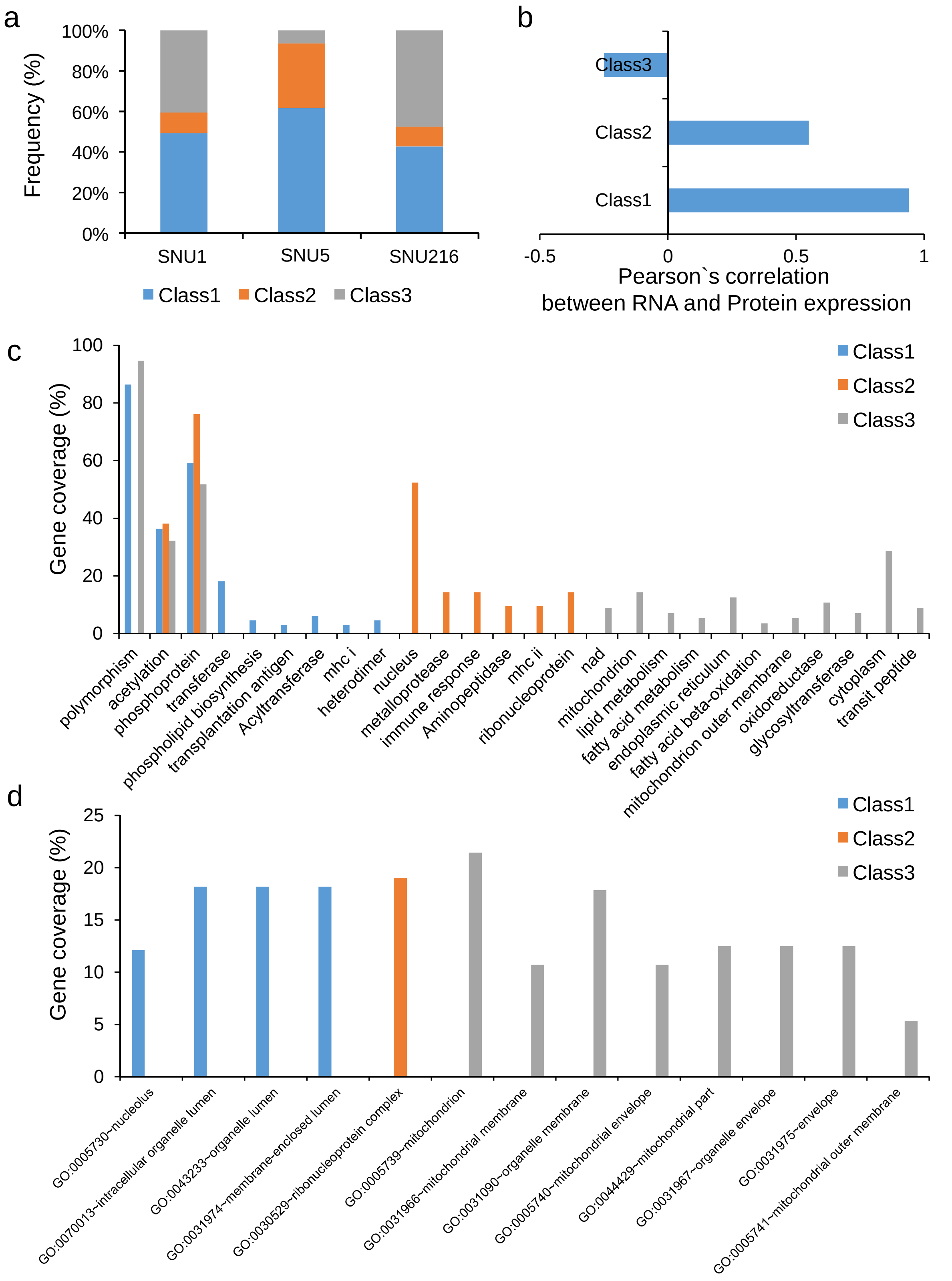

Supplementary Figure S5 Characterization of nonsynonymous variants. (a) Distribution of different class of variants. (b) The correlation between mRNA and protein in the proportion of variant form.

$RPKM_B/(RPKM_A + RPKM_B)$ vs. $PSM_B/(PSM_A + PSM_B)$ where A refers to reference form and B refers to variant form. (c-d) Classification of variant genes according to 'Protein Information Resource' (c) and 'Cellular Component' (d) terms ($P < 0.05$).

B4E3E9.LLDTAFDLDVFK

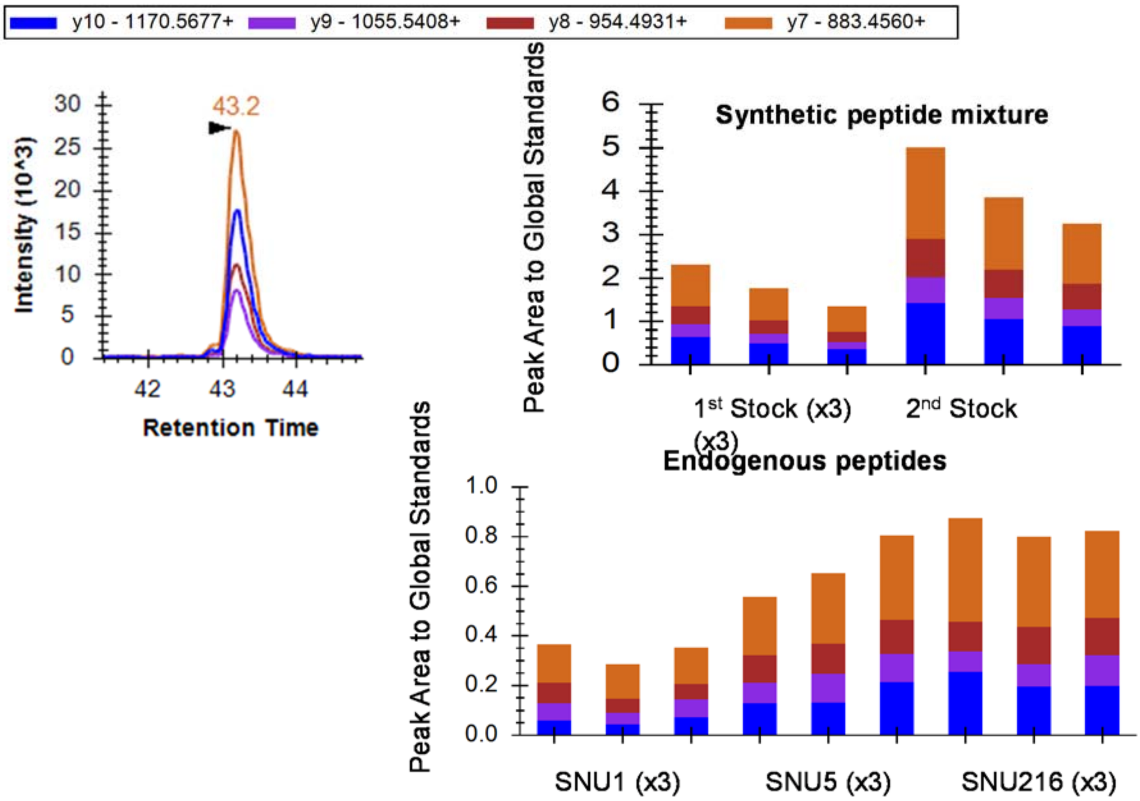

B4E3E9_v1.LLDTAFDLDIFK

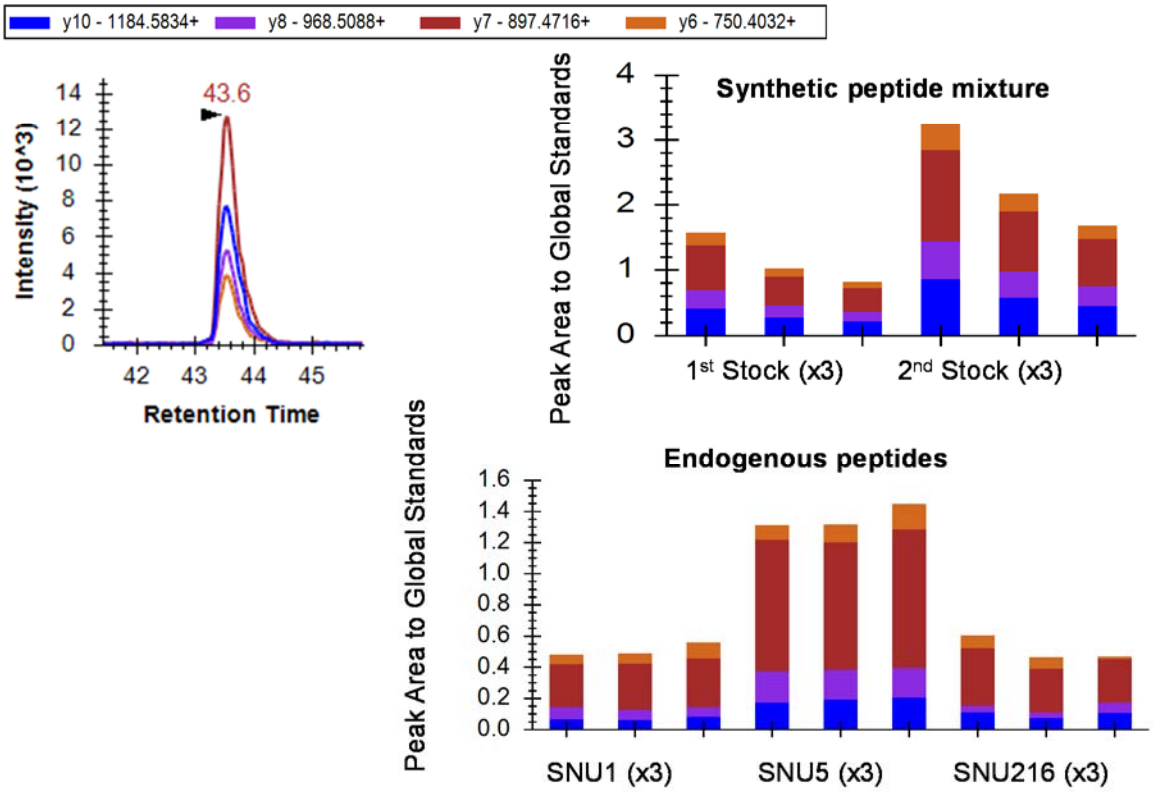

Q53EZ4.YSTTTLLEQLEETTR

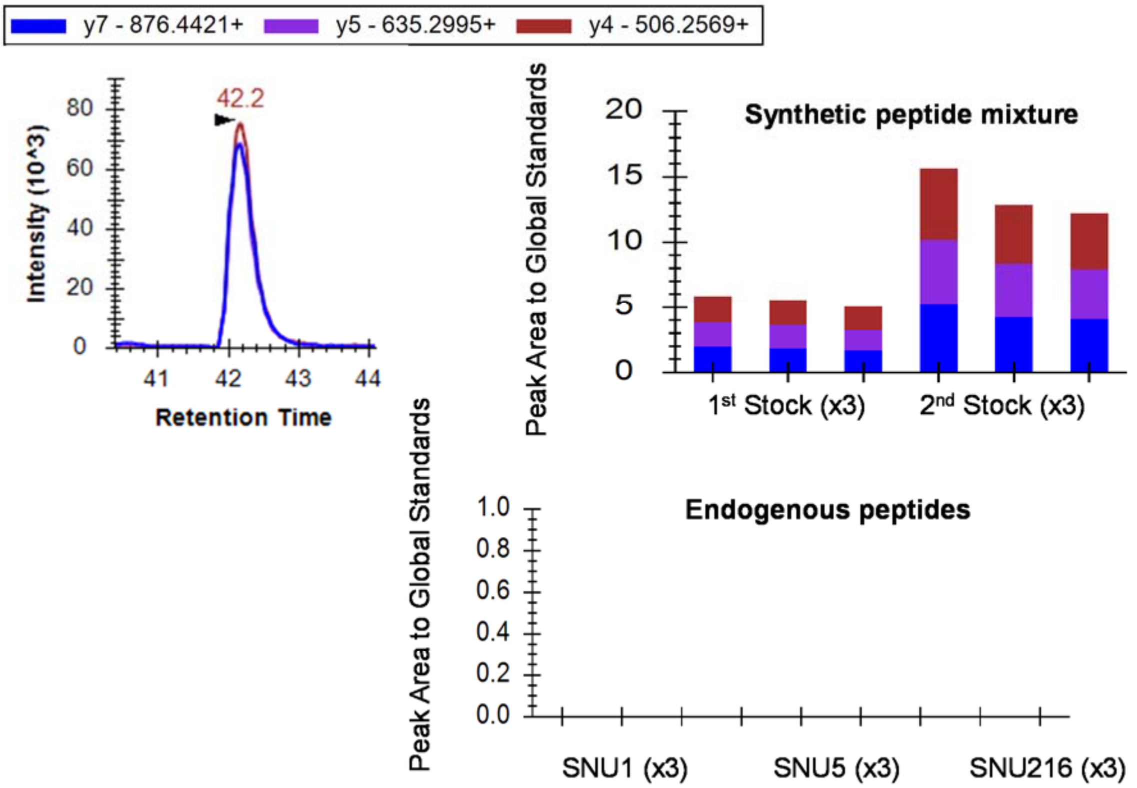

Q53EZ4_v1.YSTTALLEQLEETTR

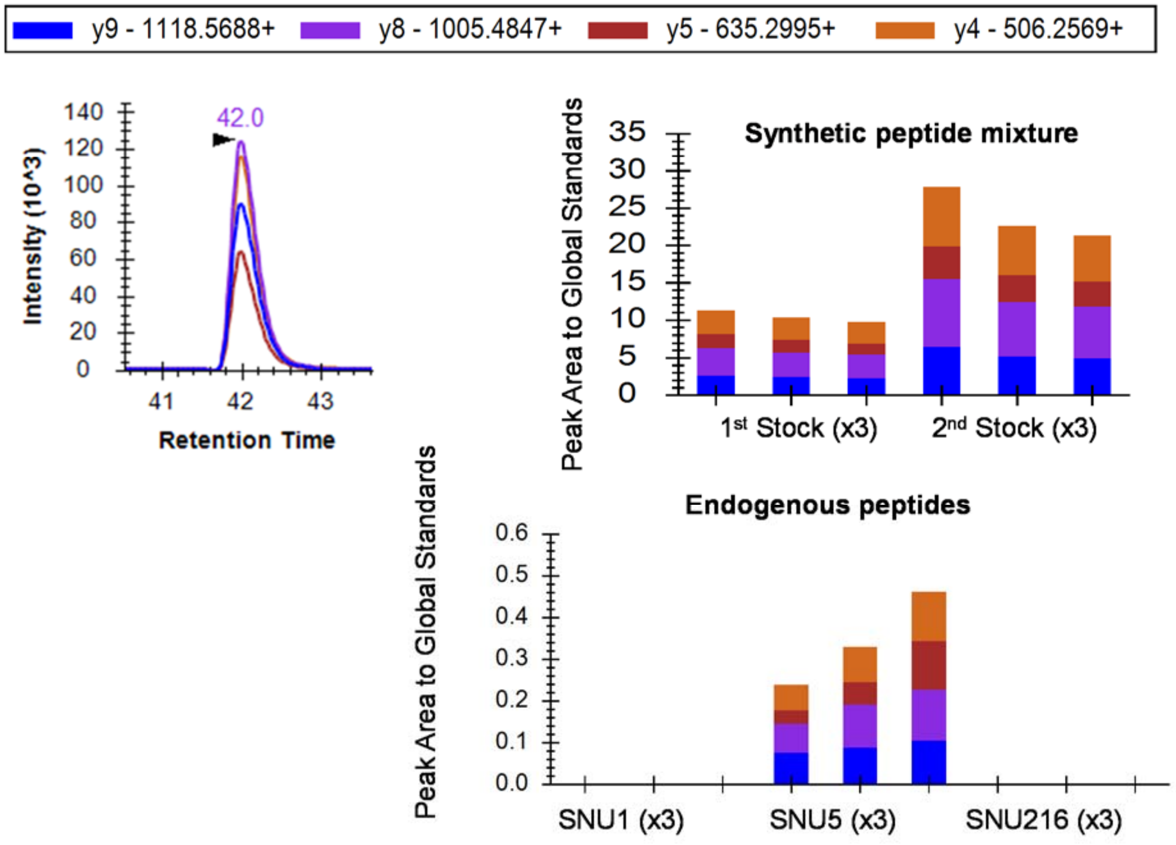

E9PBJ5.TLNEADCATVPPAIR

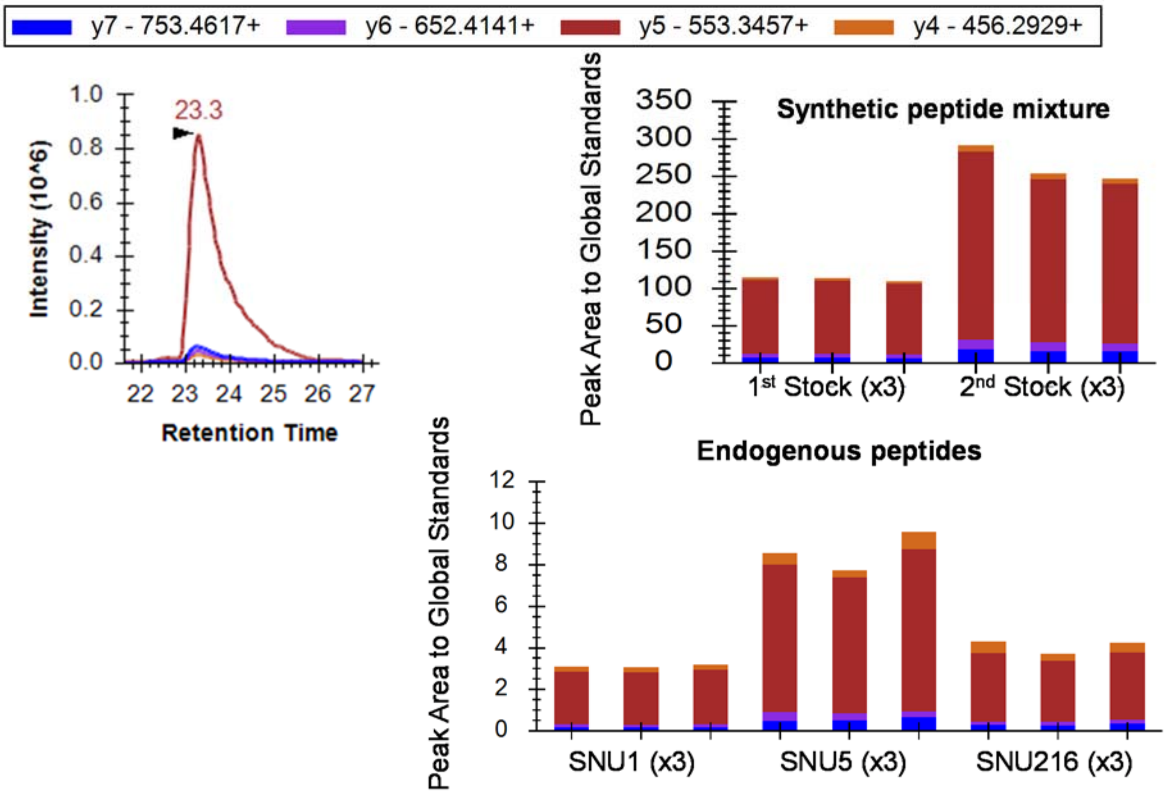

E9PBJ5_v1.TLNEADCATIPPAIR

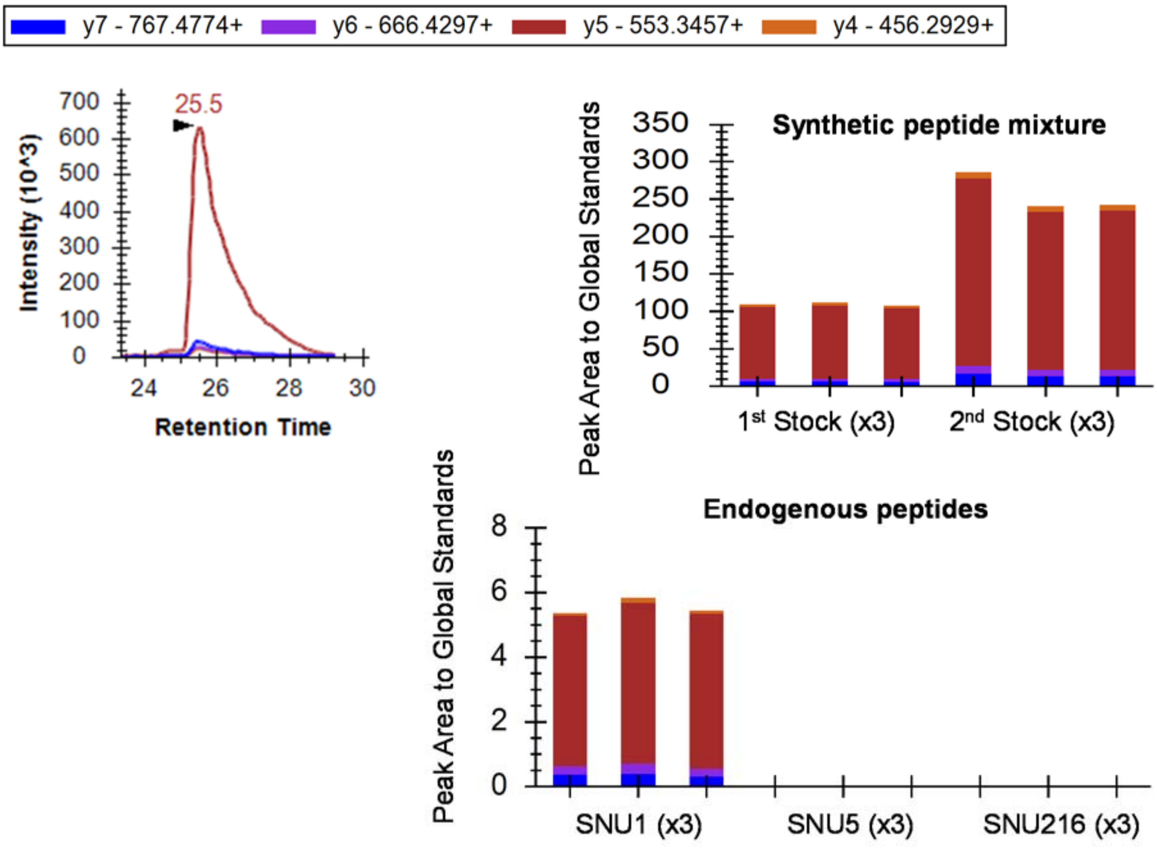

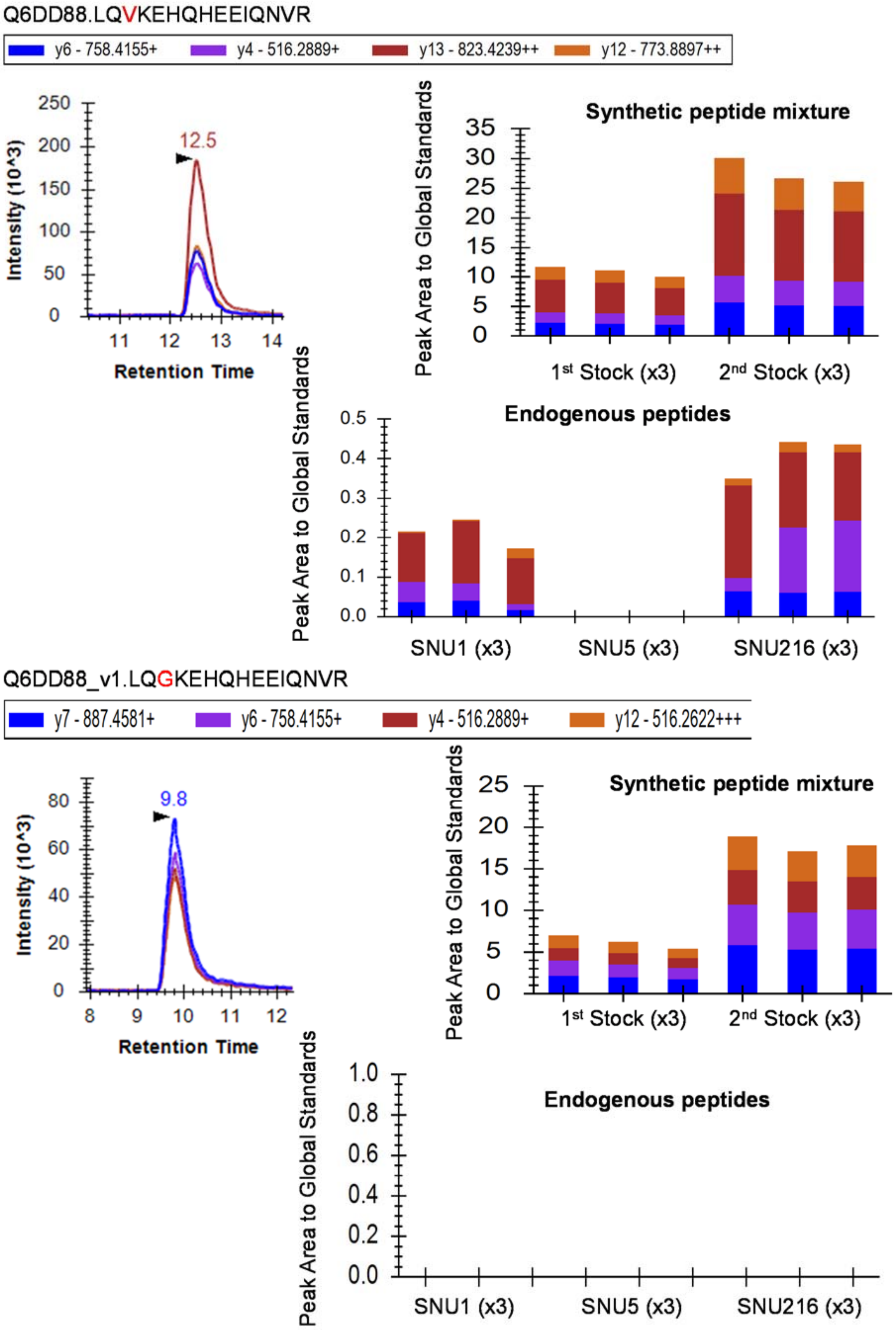

Q6DD88.LQVKEHQHEEIQNVR
y6 - 758.4155+
y4 - 516.2889+
y13 - 823.4239++
y12 - 773.8897++
Intensity (10^3)
12.5
Retention Time
Synthetic peptide mixture
Peak Area to Global Standards
1st Stock (x3)
2nd Stock (x3)
Endogenous peptides
SNU1 (x3)
SNU5 (x3)
SNU216 (x3)
Q6DD88_v1.LQGKEHQHEEIQNVR
y7 - 887.4581+
y6 - 758.4155+
y4 - 516.2889+
y12 - 516.2622+++
Intensity (10^3)
9.8
Retention Time
Synthetic peptide mixture
Peak Area to Global Standards
1st Stock (x3)
2nd Stock (x3)
Endogenous peptides
Peak Area to Global Standards
SNU1 (x3)
SNU5 (x3)
SNU216 (x3)

Q86XZ4.NFAPGETPAAIANSSGQPYQPLR

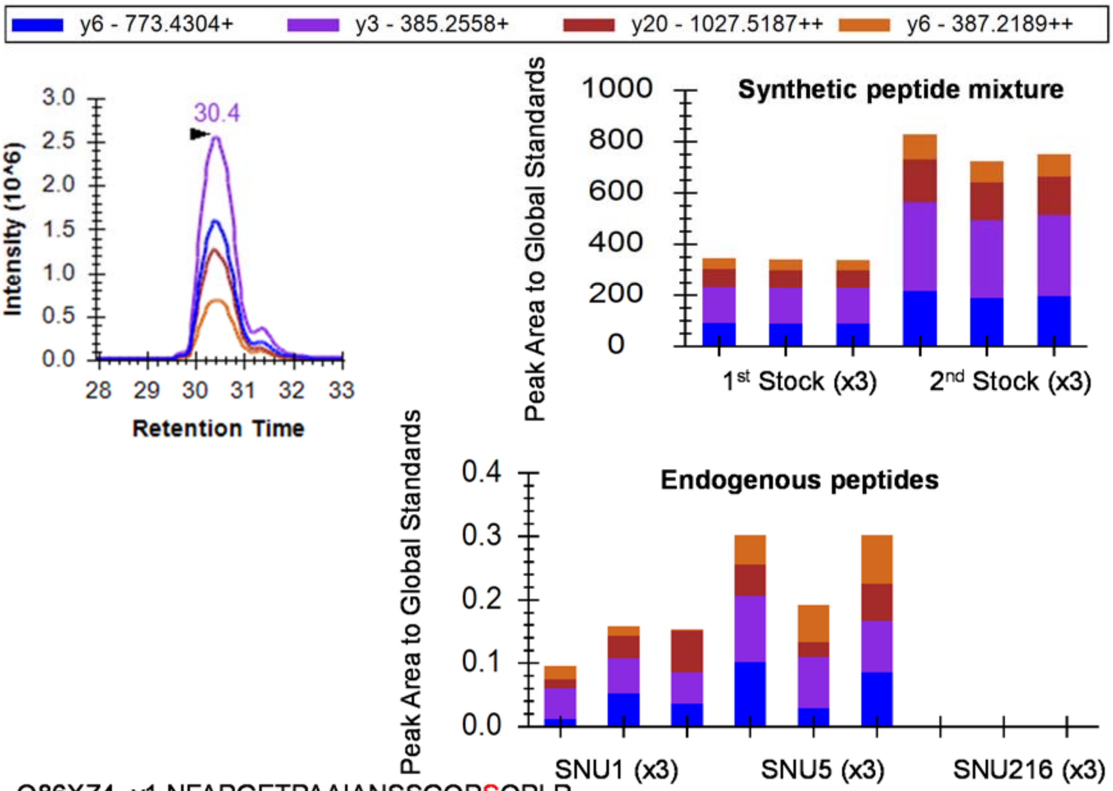

Q86XZ4_v1.NFAPGETPAAIANSSGQPSQPLR

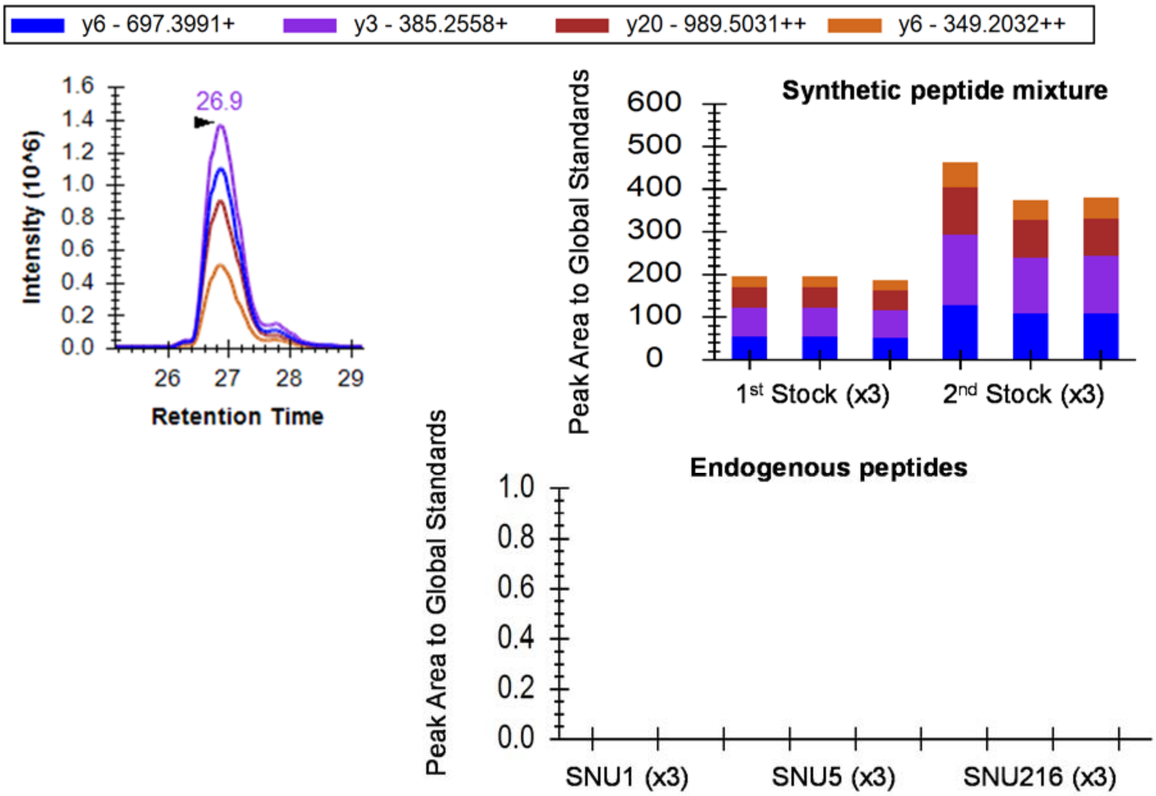

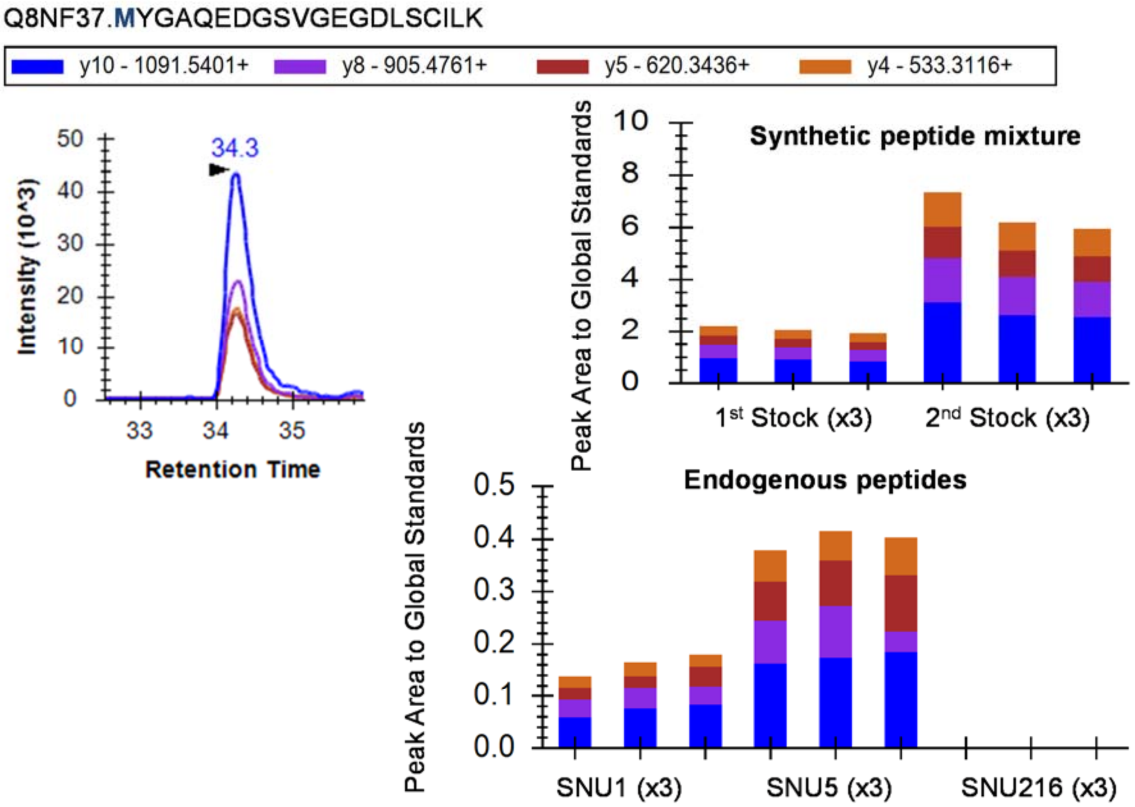
Q8NF37.MYGAQEDGSVGEGDLSCILK
y10 - 1091.5401+
y8 - 905.4761+
y5 - 620.3436+
y4 - 533.3116+
34.3
Intensity (10^3)
Retention Time
Peak Area to Global Standards
Synthetic peptide mixture
1st Stock (x3)
2nd Stock (x3)
Endogenous peptides
SNU1 (x3)
SNU5 (x3)
SNU216 (x3)

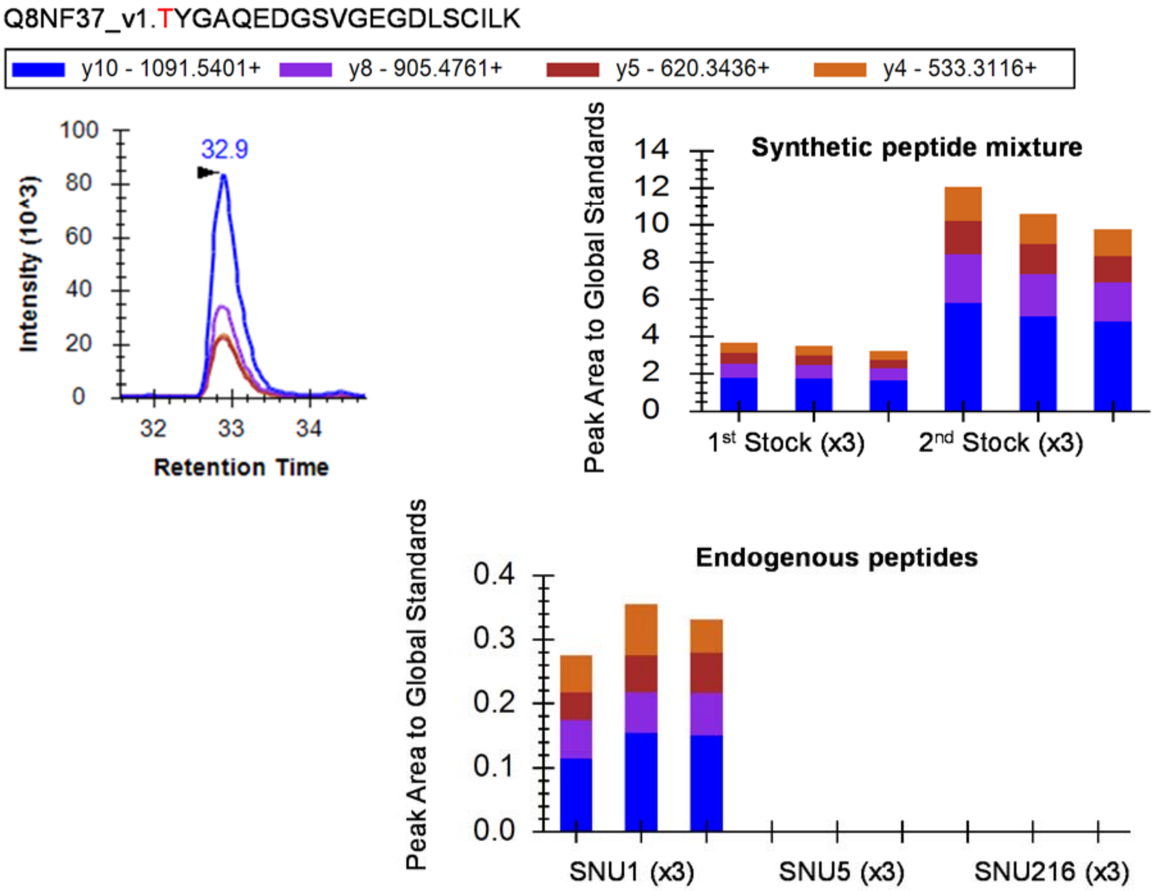
Q8NF37_v1.TYGAQEDGSVGEGDLSCILK
y10 - 1091.5401+
y8 - 905.4761+
y5 - 620.3436+
y4 - 533.3116+
32.9
Intensity (10^3)
Retention Time
Peak Area to Global Standards
Synthetic peptide mixture
1st Stock (x3)
2nd Stock (x3)
Endogenous peptides
SNU1 (x3)
SNU5 (x3)
SNU216 (x3)

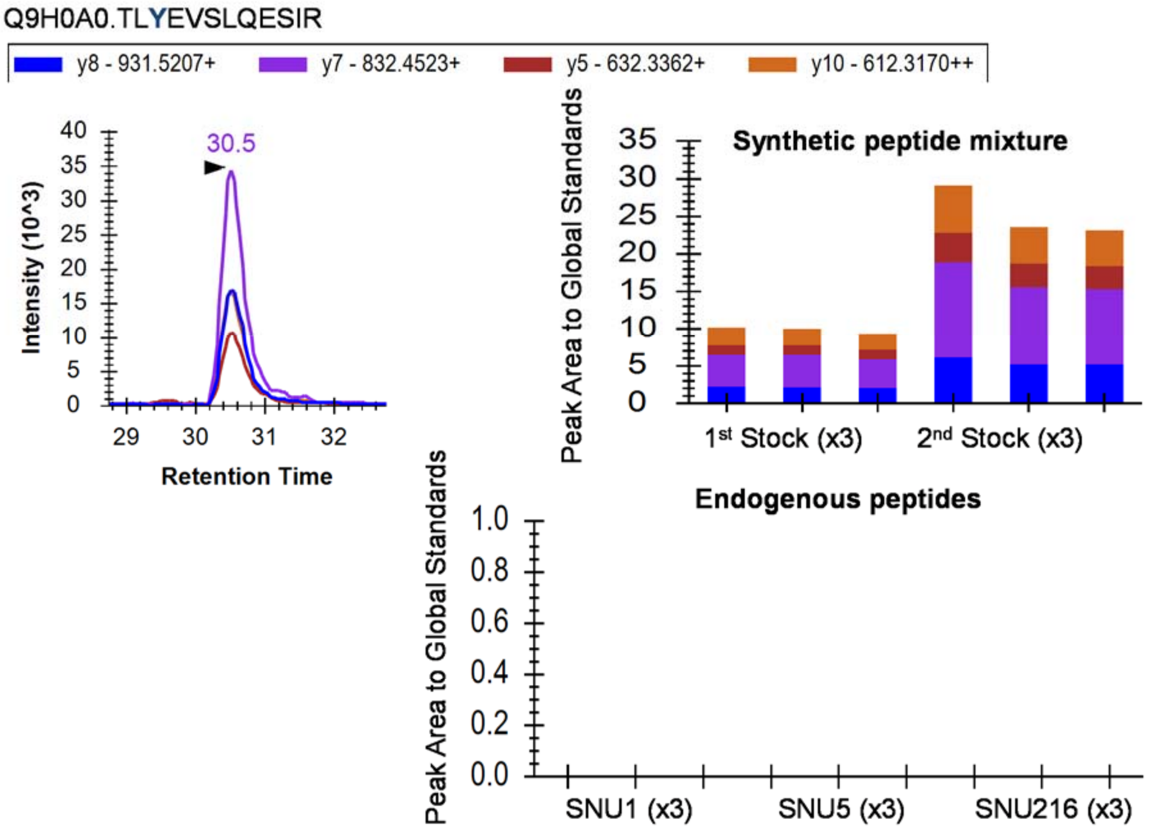

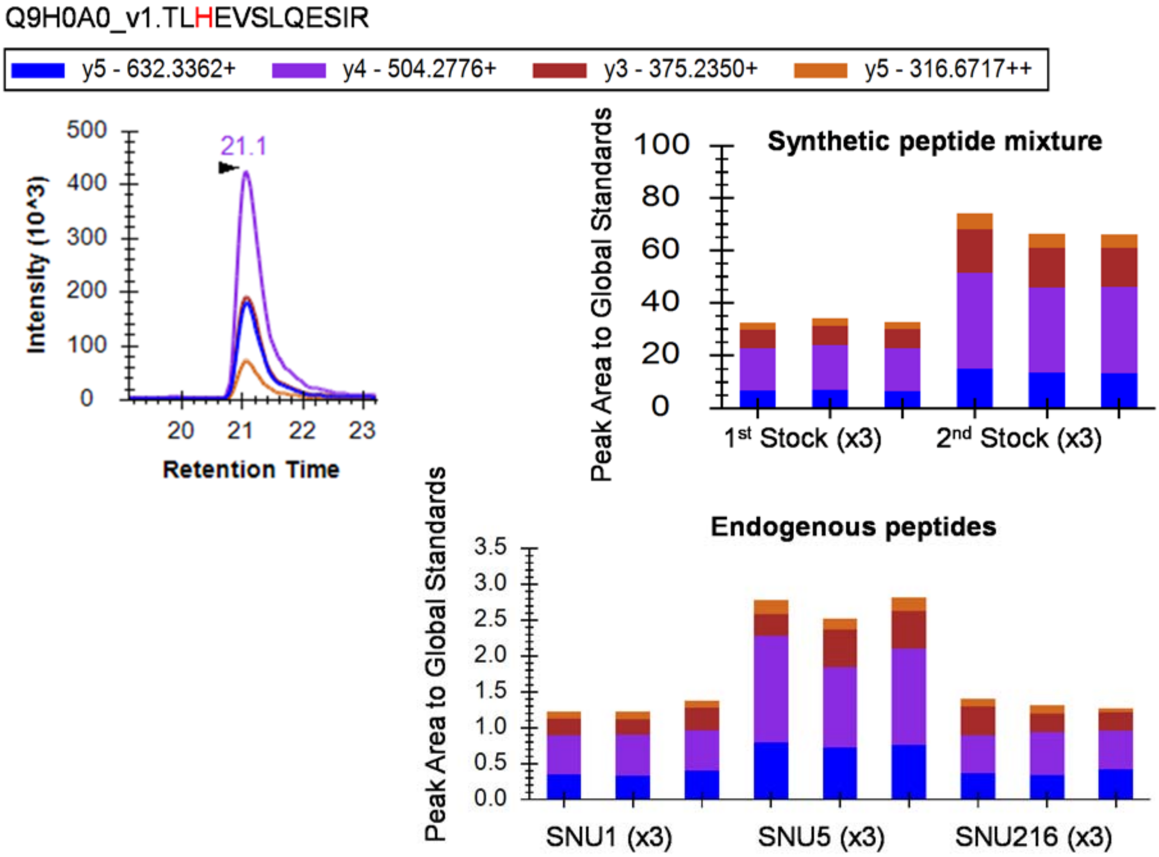

Supplementary Figure S6 LC-MRM validation of 14 target peptides using the corresponding synthetic

peptides. Each of the synthetic peptides are represented by XICs of all MRM transitions. Relative peak areas of the standard peptides and the endogenous peptides are presented.

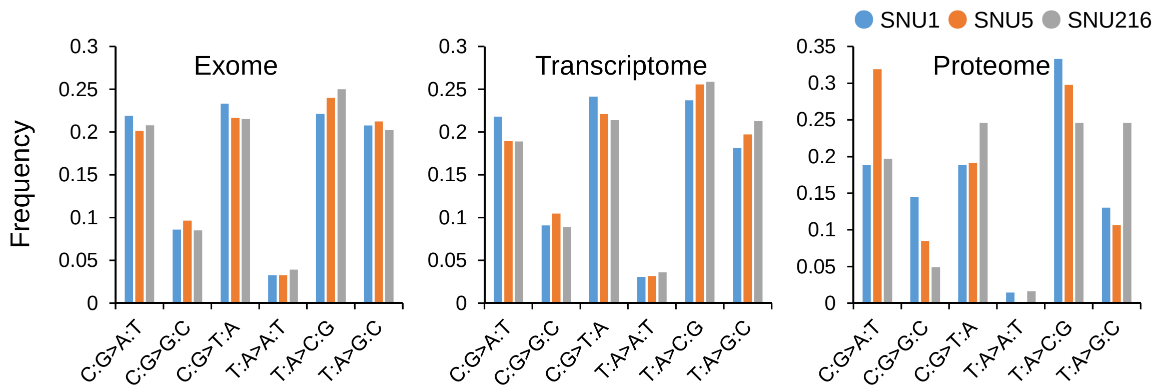

Supplementary Figure S7 Classification of variants based on the type of genetic change. For mRNA and protein, only the detected variants are presented. The frequency of each genotype did not change significantly by the cell type or dataset. The result shows an enrichment at C:G>A:T, C:G>T:A, T:A>C:G and T:A>G:C type.

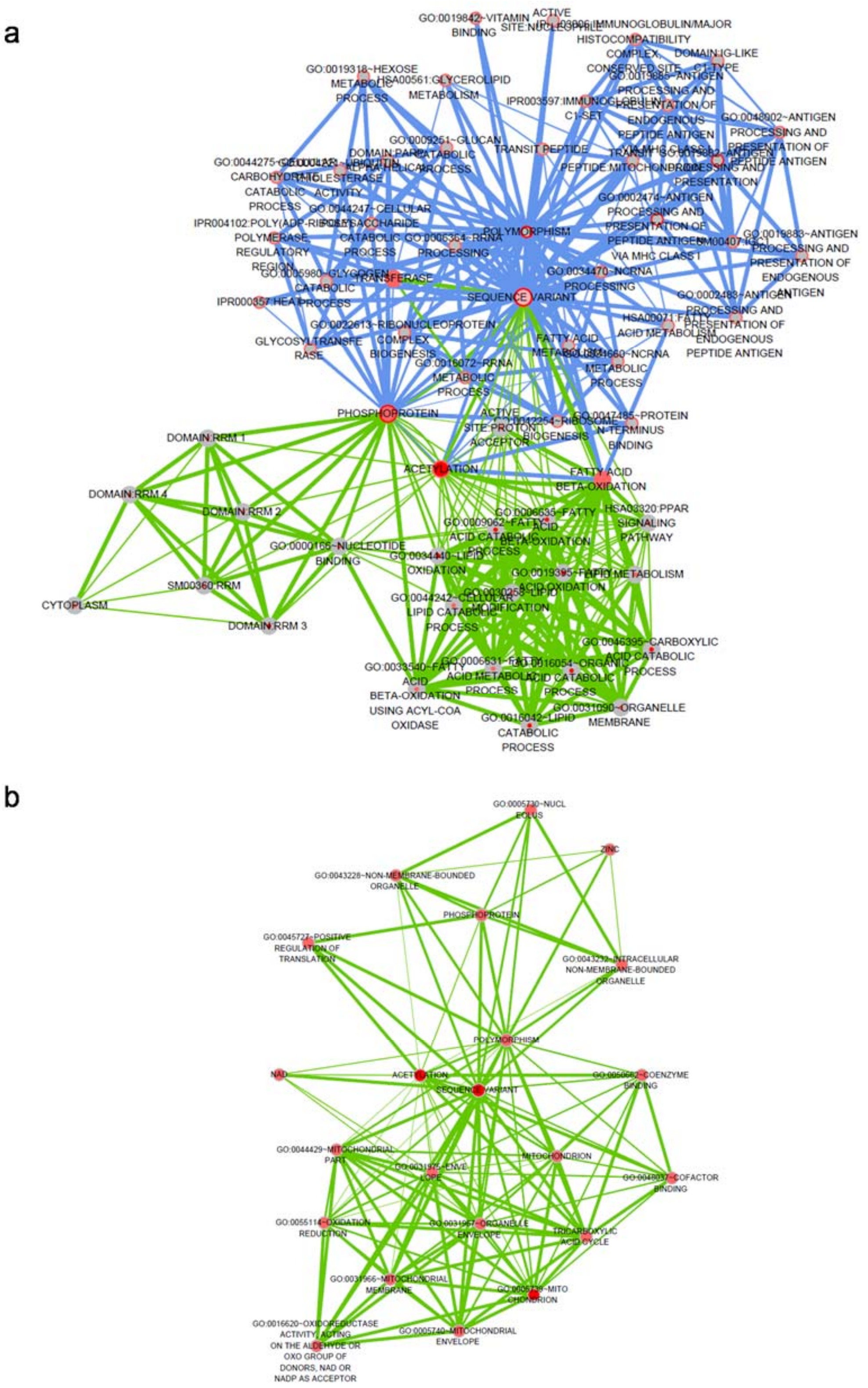

Supplementary Figure S8 Functional annotation of variant genes using the Enrichment map. (a) Variant genes are differentially coded by their protein-level allelic types (P < 0.05); AA type (inner circle, green line) versus BB type (outer circle, blue line). “Acetylation” is significantly enriched

functional term in AA type dataset and “polymorphism” in BB type dataset. (b) Functional annotation of AB type variant genes. Top functional enrichment terms of AB type are “acetylation”, “polymorphism” and “sequence variant”. Node size indicates the number of genes in each dataset.
